# Real-World Evidence of Multiple Air Pollutants and Mortality: A Prospective Cohort Study in an Oldest-Old Population

**DOI:** 10.1021/envhealth.3c00106

**Published:** 2023-11-09

**Authors:** Linxin Liu, Yi Zeng, John S. Ji

**Affiliations:** † Vanke School of Public Health, Tsinghua University, Beijing, China 100084; ‡ School of Medicine, Tsinghua University, Beijing, China 100084; § Center for the Study of Aging and Human Development, School of Medicine, Duke University, Durham, North Carolina 27710, United States; ∥ Center for Healthy Aging and Development Studies, National School of Development, Peking University, Beijing, China 100091

**Keywords:** air quality, multiple exposures, climate, district economic, health equity

## Abstract

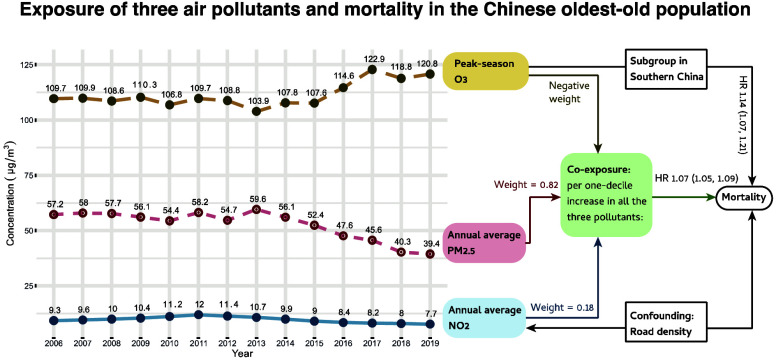

We aimed to report real-world longitudinal ambient air pollutants levels compared to WHO Air Quality Guidelines (AQG) and analyze multiple air pollutants’ joint effect on longevity, and the modification and confounding from the climate and urbanization with a focus on the oldest-old. This study included 13,207 old participants with 73.3% aged 80 and beyond, followed up from 2008 to 2018 in 23 Chinese provinces. We used the Cox-proportional hazards model and quantile-based g-computation model to measure separate and joint effects of the multiple pollutants. We adjusted for climate and area economic factors based on a directed acyclic graph. In 2018, no participants met the WHO AQG for PM_2.5_ and O_3_, and about one-third met the AQG for NO_2_. The hazard ratio (HR) for mortality was 1.07 (95% confidence interval-CI: 1.05, 1.09) per decile increase in all three pollutants, with PM_2.5_ being the dominant contributor according to the quantile-based g-computation model. In the three-pollutant model, the HRs (95% CI) for PM_2.5_ and NO_2_ were 1.27 (1.25, 1.3) and 1.08 (1.05, 1.12) per 10 μg/m^3^ increase, respectively. The oldest-old experienced a much lower mortality risk from air pollution compared to the young-old. The mortality risk of PM_2.5_ was higher in areas with higher annual average temperatures. The adjustment of road density considerably intensified the association between NO_2_ and mortality. The ambient PM_2.5_ and O_3_ levels in China exceeded the WHO AQG target substantially. Multiple pollutants coexposure, confounding, and modification of the district economic and climate factors should not be ignored in the association between air pollution and mortality.

## Introduction

According to the World Health Organization (WHO), 99% of the world’s population lives in places where air pollution levels exceed WHO guideline limits. The WHO introduced an updated air quality guideline (AQG) in 2021 and established new interim targets for common air pollutants, such as fine particulate matter (PM_2.5_), nitrogen dioxide (NO_2_), ozone (O_3_), sulfur dioxide, and carbon monoxide,^1^ based on systematic reviews of mortality risks in the target pollutants.^2,3^ It lowered the recommended annual average concentration of PM_2.5_ from 10 to 5 μg/m^3.^^1^ Since most studies included in the guideline meta-analyses and systematic reviews are from North America and Europe, where air pollution mixtures and exposure ranges differ from other regions, this will result in uncertainty in concentration–response curves.^4^ The annual mean levels of PM_2.5_ and the mortality rate attributed to ambient air pollution in China were much higher than Northern America and Europe.^5^ The United Nations’ Sustainable Development Goals (SDGs) report in 2022 evaluated China’s progress toward SDGs 3 and 11 regarding air pollution and related health as “significant challenges remain”.^6^

Air pollution and mortality longitudinal studies have emerged in China and mainly focused on the separate association of a single pollutant and health outcome or only adopted multiple-pollutant model for mutual adjustment.^7,8^ Few air pollutants occur in isolation, so a better understanding of air pollution on premature mortality must take the synergistic effects of pollutant mixtures and coexposures of correlated multiple pollutants into account to establish causal relationships. While some large cohort studies in developed countries suggest PM_2.5_, O_3_, and NO_2_ were causally associated with a greater mortality risk and that PM_2.5_ posed the greatest risk,^9^ systematic reviews indicate the quality of evidence for the long-term NO_2_ and O_3_ with all-cause mortality risk is considered moderate.^2,3^ Furthermore, our previous findings showed that NO_2_’s association with mortality was explained by PM_2.5._^10^ It is worth noting that NO_2_ exhibits a positive correlation with road traffic, which serves as an economic indicator in developing countries like China. However, there is a notable absence of adjustments for these potential district-level confounders in studies investigating the relationship between air pollutants and health.

There have been limited cohorts specifically targeting the oldest-old (aged 80 years and above) population, particularly centenarians. The vast geographic and demographic diversity in China also provide opportunities to investigate various vulnerable populations and district-level disparities related to environmental health. A previous study found per capita NOx emission inequality increased from 2006 to 2015 in China.^11^ The author then conducted an ecological study that demonstrated that this air pollution inequality impacted health inequality based on 31 Chinese provinces.^12^ Despite these data at the macro level, we need real-world evidence at the individual level for better understanding and improvement in air pollution health disparity.

First, our study utilized a longitudinal cohort study of China’s oldest-old population to investigate the temporal and spatial variations of three air pollutants: PM_2.5_, NO_2_, and O_3_. Second, we hypothesized that these three air pollutants would exhibit positive associations with mortality risk and used multiple air pollutants models to calculate the separate and joint health effects of air pollutants on mortality. Third, we aimed to identify vulnerable populations to air pollution among those with different demographic characteristics, lifestyles, and comorbidities, with an emphasis on the oldest-old group. Furthermore, we examined the possible confounding and modification of the area-level factors.

## Materials and Methods

### Study Population

We used the Chinese Longitudinal Healthy Longevity Survey (CLHLS). The CLHLS project has the largest sample of centenarians in the world based on our knowledge (plus compatible groups of nonagenarians, octogenarians, and young-old aged 65–79). The detailed cohort design has been described previously.^13^ It is a longitudinal cohort designed to study longevity and aging. The survey was conceived initially to study the senior population’s socioeconomic characteristics, family, lifestyle, and demographic profile. The CLHLS adopted a targeted random-sample design to ensure representativeness, even distribution across age and sex, and sufficient subsample size of the oldest-old aged 80+, plus compatible young-old aged 65–79. Since the 2002 wave, the CLHLS was expanded from recruiting only the oldest-old in 1998 and 2000 waves to also interviewing approximately three randomly selected nearby elders aged 65–79 of predefined age and sex in conjunction with every two centenarians.^14^ The questionnaire design was based on international standards and was adapted to the Chinese cultural/social context and carefully tested by pilot studies and interviews.^14^ Analyses of health measures showed high reliability and validity on evaluated items and exceeded widely used criteria.^15^ We used the 2008/2009 cohort of CLHLS with urban and rural coverage in 23 provinces. The participants were enrolled in 2008 or 2009 and followed up to 2018 roughly every two years. We overlaid environmental exposure data based on the residential area with remote sensing. We included 13,207 participants after excluding 3,747 participants from the 2008/2009 cohort (see Supplementary Methods in the Supporting Information). The average age (87 vs 86), proportion of females (58% vs 55%), annual average O_3_ exposure (92 vs 92 μg/m^3^), and NO_2_ exposure (19 vs 17 μg/m^3^) were similar between the included and excluded groups. The included participants had more participants without schooling (64% vs 55%), and more participants living in rural areas (64% vs 50%), and higher annual average PM_2.5_ exposure (53 vs 48 μg/m^3^) than the excluded participants.

The research ethics committees of Peking University (IRB00001052-13074) and Duke University approved the study. All participants in the study have given informed consent.

### Air Pollutant Exposure Assessment

We used the annual PM_2.5_ data at 0.01° × 0.01° calculated by the Atmospheric Composition Analysis Group. They estimated the ground-level PM_2.5_ for 1998–2020 (V5.GL.02) by combining Aerosol Optical Depth (AOD) retrievals from the NASA MODIS, MISR, and SeaWIFS instruments with the GEOS-Chem chemical transport model and subsequently calibrating to global ground-based observations using a Geographically Weighted Regression (GWR), as detailed in ref (16). Annual mean PM_2.5_ estimates exhibit a general consistency with ground-based observations with the coefficient of determination (*R*^2^) as 0.68–0.91 for Asia.^16^ The annual concentrations of nitrogen dioxide (NO_2_) concentration levels (μg/m^3^) were obtained at one-kilometer spatial resolution using a land-use regression model corrected for satellite pass time and cloud coverage.^17^ The land-use regression model was corrected for satellite pass time, cloud coverage was directly used for urban areas, and model performance differed regionally with *R*^*2*^ as 0.52 in Asia. For rural areas, NO_2_ concentrations were adjusted using surface NO_2_ concentrations derived from the Ozone Monitoring Instrument satellite NO_2_ columns, and the correlation between the estimated surface concentrations and ground measurements is improved from Pearson’s correlation coefficient (*r*) of 0.51 in the original product to 0.58.^18,19^ A nationwide daily maximum 8-h average (MDA8) at a resolution of 0.1° × 0.1° prediction model based on the eXtreme Gradient Boosting (XGBoost) algorithm was established by combining the MDA8 ozone observations from 2013 to 2017 with concurrent ozone retrievals, aerosol reanalysis, meteorological parameters, and land-use data.^20^ External model testing *R*^*2*^ ranged from 0.60 to 0.87 at the month level in different years.^20^ We matched the air pollutants concentrations to the residence of the participants. We calculated annual ambient PM_2.5_, NO_2_, O_3_ and the last peak-season (May to September) ambient O_3_ concentration the participants experienced.

### Mortality Outcome Assessment

The immediate family members of the subjects reported the mortality information in the follow-up surveys. The date of death would be validated by death certificates when available. We used all-cause mortality. We measured the survival time in months from the first interview to the recorded death date or last interview date.

### Climate-Related Factors Measurement

Daily meteorological data of the weather monitoring stations across China between 2008 to 2018 was obtained from the China Meteorological Administration. Each study participant was matched with meteorological data collected from a monitoring station closest to their residence. We used the annual average and standard deviation of the daily mean temperature within the year as the two variables in our analyses. The season of the death or last survey month was spring (March–May), summer (June–August), fall (September–November), and winter (December–February). The elevation data are from SRTM 90m DEM Digital Elevation Database.^21^

We divided the geographical region based on residential location to account for climate and dietary differences: central China (Henan, Anhui, Jiangxi, Hubei, Hunan), eastern China (Shandong, Shanghai, Jiangsu, Zhejiang, Fujian), northeastern China (Heilongjiang, Jilin, and Liaoning), northern China (Beijing, Tianjin, Hebei, Shanxi, Shaanxi), southern China (Guangdong, Guangxi, and Hainan), and southwestern China (Chongqing and Sichuan).

### Area Economic Measurement

We followed the CLHLS residence categories: “Urban” (including “City” and “Town”) and “Rural”. We defined the road density as the sum of road length within 5 km buffer around the residence. We filtered highway categories “motorway”, “trunk”, “primary”, “secondary”, “tertiary”, and “residential” in the China street map in 2022 from OpenStreetMap (OSM) to calculate the weighted road length sum:
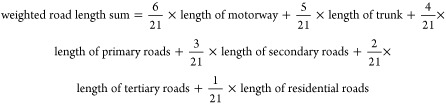


The city-level GDP per capita (yuan) and population number per kilometer in 2008 were from the CNKI China Economic and Social Big Data Research Platform.

### Other Covariates and Modifiers Measurements

We considered baseline characteristics, including age, sex, marital status, education, smoking status, drinking status, physical activity, household income, coresidence, housework frequency, social activity frequency, Mini-Mental State Examination (MMSE), Activities of Daily Living (ADL) disability and Body Mass Index (BMI). The detailed definition is shown in the Supplementary Methods (see the Supporting Information).

### Statistical Analysis

Given the open cohort nature of our cohort with various subjects contributing different person-times to the analysis, we decided to use the Cox proportional hazards model to examine the association between long-term PM_2.5_, NO_2_, and O_3_ exposure and all-cause mortality. We ran a series of models to test the robustness of the association. The first model was the unadjusted model (Model A), and then it was gradually adjusted for potential confounders based on the Directed Acyclic Graph (DAG) (Figure S1) or predictors of the outcome: age, sex, education, household income, marriage, coresidence, exercise, smoking, alcohol drinking, housework frequency, social activity frequency, residence (Model B), region, temperature annual mean, temperature annual SD, elevation, season of death (Model C), road density, city GDP per capita, city population density (Model D), BMI, MMSE score, and ADL score (Model E). The air pollutants were further mutually adjusted in the multiple pollutant model (Model F to K). Considering the possible multicollinearity of the three pollutants and to assess the joint effect of the pollution mixture on mortality, we used the quantile-based g-computation model.^22^ Quantile-based g-computation estimates the overall mixture effect with the same procedure used by a weighted quantile sum (same formula as below), but estimates the parameters of a marginal structural model, rather than a standard regression.
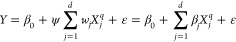


ψ is equivalent to the g-computation estimator^23^ of a joint marginal structural model for quantized exposures, which estimates the effect of increasing every exposure simultaneously by one quantile. *w*_*j*_ are the weights for each exposure (here we have *d* exposures). ε is the error term. *X*_*j*_^*q*^ is the quantized version of the *j*th exposure. *β*_*j*_ is the effect size for exposure *j*.

To assess for nonlinearity, we used the restricted cubic spline to describe the concentration–response relationship. Susceptible population and area disparity were tested via stratified analyses. We further examined the association between the last two years and three years air pollution exposure and mortality for sensitivity analysis. We used R version 4.2.1 to do all the analysis.

## Results

We included 13,207 participants with a mean age of 87 (SD: 11), and 7634 (57.8%) were females. The centenarian group consisted of 81.3% females (*n* = 2119). A total of 9245 deaths occurred during the 61,082 person-year follow-up period. Most participants did not receive a formal education (64.1%), did not live with a married spouse (69.4%), lived with family (83.4%), reported a lack of or inability to undertake regular exercise (60.9%) or social activities (89.2%), were never smokers (66.7%), or never alcohol drinker (69.2%), resided in rural areas (63.9%), or did not have disabilities (78.7%) (Tables 1 and S1). The oldest-old had a slightly higher exposure to PM_2.5_ and NO_2_ than the young-old. Their residential areas were also characterized by fewer roads and lower GDP per capita. Additionally, they had decreased engagement in housework and social activities, and a higher prevalence of ADL disability and cognitive impairment than their counterparts (Table S1).

**Table 1:** Population Characteristics^a^

variable	total (*N* = 13207)	variable	total (*N* = 13207)
**Age: Mean (SD)**	87.2 (11.4)	**Residence**	
**Sex**		Urban	4767 (36.1%)
Male	5573 (42.2%)	Rural	8440 (63.9%)
Female	7634 (57.8%)	**Region**	
**Education**		Central	3056 (23.1%)
0 year	8464 (64.1%)	Eastern	4058 (30.7%)
1–6 years	3537 (26.8%)	Northeastern	938 (7.1%)
>6 years	1206 (9.1%)	Northern	705 (5.3%)
**Marriage**		Southern	2710 (20.5%)
Married and living with a spouse	4044 (30.6%)	Southwestern	1740 (13.2%)
Not living with a spouse	9163 (69.4%)	**Road density (meters within 5km radius): Median [P25, P75]**	896 [330, 2001]
**Regular exercise**		**GDP per capita in 2008 (yuan): Median [P25, P75]**	21000 [13669, 45242]
Current	3529 (26.7%)	**Population density in 2008 (per square kilometer): Median [P25, P75]**	529 [336, 757]
Former	1641 (12.4%)	**Temperature annual mean (Celsius): Mean (SD)**	164 (40.4)
Never	8037 (60.9%)	**Temperature annual SD (Celsius): Mean (SD)**	90.7 (20.7)
**Smoking**		**Elevation (meter): Median [P25, P75]**	59 [23, 204]
Never	8809 (66.7%)	**Average PM**_**2.5**_**of the last year (μg/m**^**3**^**): Mean (SD)**	52.8 (15.8)
Former	2088 (15.8%)	**Average NO**_**2**_**of the last year (μg/m**^**3**^**): Median [P25, P75]**	16.2 [10.5, 23.1]
Light smoker	1817 (13.8%)	**Peak season O**_**3**_**last experienced (μg/m**^**3**^**): Mean (SD)**	110 (21.2)
Heavy smoker	493 (3.7%)	**Average O**_**3**_**of the last year (μg/m**^**3**^**): Mean (SD)**	92.0 (11.9)
**Drinking alcohol**			
Never	9138 (69.2%)		
Former	1850 (14.0%)		
Moderate drinker	835 (6.3%)		
Heavy drinker	1384 (10.5%)		

a Abbreviations: SD – standard deviation, P25 – percentile 25, P75 – percentile 75, GDP – Gross Domestic Product.

Geographical variations in air pollution levels were observed in China (Table S2). The annual ambient average PM_2.5_ was the highest in places with heavy industry or colder climates, such as central China, northern China, places with the highest annual temperature variation, or with the highest population density. The median of NO_2_ was higher in northern and northeastern China, urban areas, among individuals with more than primary school education, around high road density, and high annual temperature variation than their counterparts. The peak season O_3_ (μg/m^3^) was highest in eastern China, places with the lowest elevation, highest GDP per capita, highest population density, lower annual average temperature, or higher annual temperature variation.

**Figure 1:**
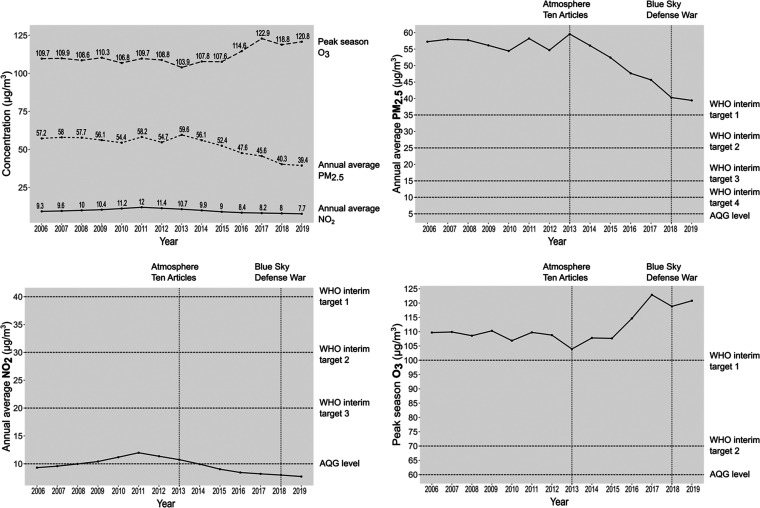
Change trend of the air pollution around the participants’ residence from 2006 to 2019. (a) Note: Abbreviation: AGQ – Air Quality Guideline. (b) In September 2013, the China State Council introduced a policy known as the “Atmosphere Ten Articles.” This policy outlined ten measures aimed at preventing and controlling air pollution including industrial restructuring, clean energy promotion, etc. In June 2018, the China State Council printed and distributed the Three-Year Action Plan for Winning the Blue Sky Defense War aiming to reduce emissions of major air pollutants and greenhouse gases, and decrease the number of days with high air pollution.

Over the decade from 2008 to 2018, the annual average PM_2.5_ (μg/m^3^) of all the participants’ residence changed from 57.2 to 39.4, peaking at 59.6 in 2013. Annual NO_2_ (μg/m^3^) changed from 9.3 to 7.7, peaked at 12.0 in 2011. Peak season O_3_ (μg/m^3^) changed from 109.7 to 120.8, peaking at 122.9 in 2017 (Figure 1). Besides, annual O_3_ (μg/m^3^) changed from 92.8 to 96.8, peaking at 100.1 in 2017. At baseline, the proportion of individuals whose exposure level met the WHO AQG was 0% for annual average PM_2.5_, 24.2% for annual average NO_2_, and 0.2% for peak season O_3_. In 2018, the proportion was still 0% for PM_2.5_, increased to 31.4% for NO_2_, and became 0% for O_3_. The average levels (SD) of PM_2.5_, NO_2_, and O_3_ in the final year (death or censored), and the nearest peak-season O_3_ were 52.8 (15.8), 19.0 (13.8), 92.0 (11.9), and 110 (21.2) μg/m^3^, respectively. Annual average PM_2.5_, NO_2_, and O_3_ were positively associated with each other, with Pearson coefficients (95% CI) of 0.37 (0.35, 0.38) for PM_2.5_ and NO_2_, 0.06 (0.04, 0.07) for PM_2.5_ and O_3_, and 0.16 (0.15, 0.18) for NO_2_ and O_3_.

The hazard ratios (HRs) for all-cause mortality of per 10 μg/m^3^ increase in PM_2.5_, NO_2_, annual average O_3_, and peak season O_3_ were 1.24 (1.22, 1.27), 1.20 (1.16, 1.24), 0.87 (0.86, 0.89), and 0.89 (0.87, 0.90), respectively, in the fully adjusted single-pollutant model (Table 2). After adjusting for the geographic region, the HR (95% CI) for PM_2.5_ showed a great increase [1.15 (1.13, 1.16) to 1.22 (1.2, 1.24)]. The HR (95% CI) of NO_2_ also increased greatly after adjusting for road density, city GDP per capita, and city population density [1.06 (1.04, 1.08) to 1.21 (1.17, 1.25)]. However, this association decreased greatly when additionally adjusted for PM_2.5_ [1.06 (1.03, 1.10)]. On the other hand, the HR for O_3_ did not change significantly and remained less than 1 in models with different adjustments (Table 2).

**Table 2:** Association between Air Pollutants and Mortality Risk^a^

model	adjustment	annual PM_2.5_	annual NO_2_	annual O_3_	peak-season O_3_
**A**	**Unadjusted**	1.24 (1.23, 1.26)	1.07 (1.05, 1.09)	0.85 (0.83, 0.86)	0.93 (0.92, 0.93)
**B**	**Adjusted for age, sex, education, household income, marriage, coresidence, exercise, smoking, alcohol drinking, housework frequency, social activity frequency, residence**	1.15 (1.13, 1.16)	1.05 (1.04, 1.07)	0.9 (0.89, 0.92)	0.95 (0.94, 0.96)
**C**	Model B additionally adjusted for region, temperature annual mean, temperature annual SD, elevation, season	1.24 (1.22, 1.26)	1.06 (1.04, 1.08)	0.88 (0.86, 0.9)	0.89 (0.88, 0.9)
**D**	Model C additionally adjusted for road density, city GDP per capita, city population density	1.25 (1.22, 1.27)	1.21 (1.17, 1.25)	0.87 (0.86, 0.89)	0.89 (0.87, 0.9)
**E**	Model D additionally adjusted for BMI, MMSE score, ADL score	1.24 (1.22, 1.27)	1.20 (1.16, 1.24)	0.87 (0.86, 0.89)	0.89 (0.87, 0.9)
**F**	Model E additionally adjusted for annual PM_**2.5**_		1.06 (1.03, 1.1)	0.88 (0.86, 0.9)	0.85 (0.84, 0.86)
**G**	Model E additionally adjusted for annual NO_**2**_	1.23 (1.2, 1.25)	\	0.88 (0.86, 0.9)	0.88 (0.86, 0.89)
**H**	Model E additionally adjusted for annual O_**3**_	1.24 (1.22, 1.26)	1.19 (1.15, 1.22)	\	\
**I**	Model E additionally adjusted for peak season O_**3**_	1.29 (1.27, 1.31)	1.23 (1.19, 1.27)	\	\
**J**	Model E additionally adjusted for annual PM_**2.5,**_**annual NO**_**2**_**, annual O**_**3**_	1.22 (1.2, 1.25)	1.05 (1.02, 1.09)	0.88 (0.86, 0.9)	\
**K**	Model E additionally adjusted for annual PM_**2.5,**_**annual NO**_**2**_**, peak season O**_**3**_	1.27 (1.25, 1.30)	1.08 (1.05, 1.12)	\	0.85 (0.84, 0.86)

a Abbreviations: SD – standard deviation, GDP – Gross Domestic Product, BMI – body mass index, MMSE – Mini-Mental State Examination, ADL – Activities of Daily Living.

The HRs in the three-pollutant model were 1.22 (1.2, 1.25), 1.05 (1.02, 1.09), 0.88 (0.86, 0.9) for PM_2.5_, NO_2_, and annual average O_3_, and 1.27 (1.25, 1.3), 1.08 (1.05, 1.12), and 0.85 (0.84, 0.86) for PM_2.5_, NO_2_, and peak season O_3_, respectively. According to the quantile-based g-computation model, the HR (95% CI) for mortality was 1.07 (1.05, 1.09) for a one-decile increase in all three air pollutants simultaneously. Both PM_2.5_ and NO_2_ had positive weights, and the weight for PM_2.5_ was considerably larger than that for NO_2_ (0.82 vs 0.18). In contrast, O_3_’s weight was negative (−1).

**Figure 2:**
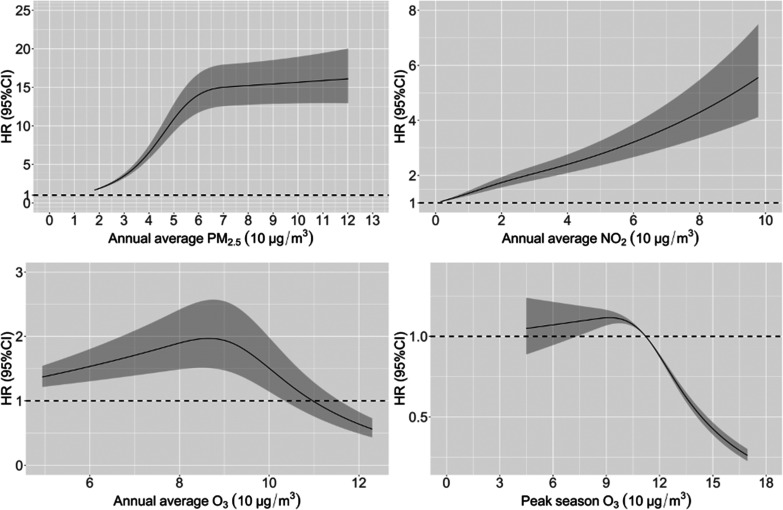
The spline for the air pollutants and mortality. Note: All the models adjusted for age, sex, education, household income, marriage, coresidence, exercise, smoking, alcohol drinking, housework frequency, social activity frequency, residence, region, temperature annual mean, temperature annual standard deviation, elevation, season of death, road density, city Gross Domestic Product per capita, city population density, body mass index, Mini-Mental State Examination score, Activities of Daily Living core.

The restricted cubic spline for PM_2.5_ was supralinear (concave downward), meaning there were larger changes in risk for low concentrations compared to higher concentrations. We identified a subtle curvature in the association between NO_2_ and mortality, with a more pronounced marginal effect occurring at higher levels of NO_2_ exposure. The HR for annual O_3_ increased at O_3_ levels lower than 88 μg/m^3^ and subsequently decreased to less than one as O_3_ levels increased beyond 110 μg/m^3^. Similarly, the HR for peak season O_3_ decreased to less than one when O_3_ was higher than approximately 110 μg/m^3^ (Figure 2). The p value for nonlinear Wald statistics were all less than 0.0001, suggesting nonlinear associations between the three pollutants and mortality.

In the stratified analysis, PM_2.5_ and NO_2_ were found to have a stronger harmful association with mortality for those under 80 years old compared to those oldest-old. There was no significant difference in HRs for females and males. Those who were married and currently living with their spouse, smoked more, drank alcohol more, reported exercising currently, doing housework every day, with higher MMSE scores, and without ADL disability had a higher mortality risk related to PM_2.5_ and NO_2_ than their counterparts (Figure S2).

**Figure 3:**
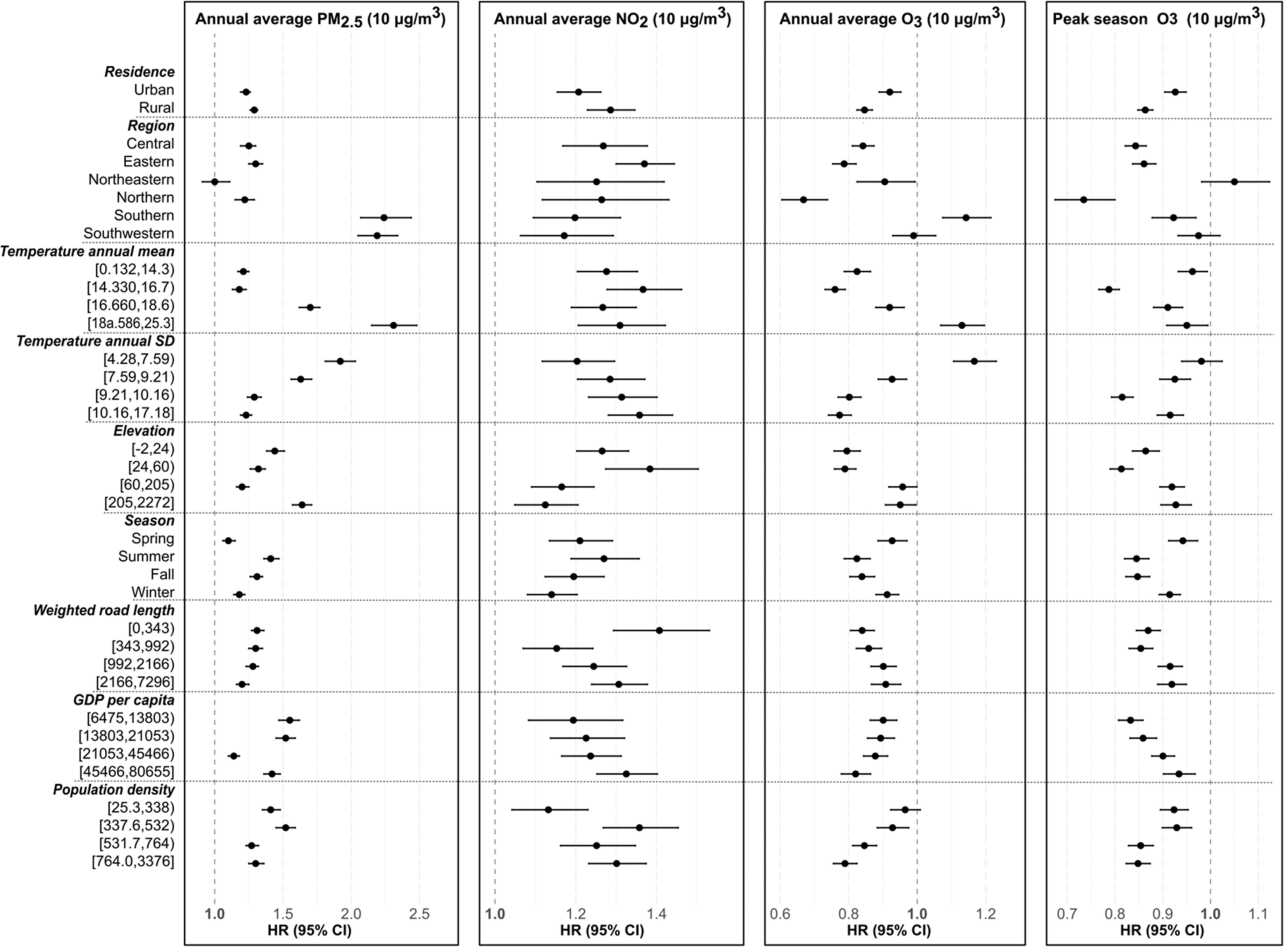
Association between air pollutants and mortality stratified by area characteristics. The unit: temperature - Celsius (°C), elevation - meter, weighted road length - meters in a 5 km radius, GDP per capita - RMB, population density - population per square kilometer.

The mortality risk associated with increasing PM_2.5_ was higher in southern China [HR (95% CI): 2.24, (2.07, 2.44)] and southwestern China [2.19 (2.05, 2.34)] than in other regions, and it tended to increase with the annual average temperature increasing and the temperature annual SD decreasing. The HR of PM_2.5_ became not statistically significant in the northeastern region [1.00 (0.91, 1.12)]. Annual O_3_ only had an HR greater than one in southern China (including Guangdong Guangxi, and Hainan) [1.14 (1.07, 1.21)] and the areas with the highest annual average temperature and the lowest temperature annual SD. The HR of NO_2_ was higher in eastern China [1.37 (1.30, 1.44)], and it increased as the temperature annual SD became larger. Additionally, PM_2.5_ had a slightly higher HR in rural areas compared to urban areas. We did not observe linear trends for the HR of PM_2.5_ or NO_2_ with the increase of road density, GDP per capita, or population density (Figure 3).

## Discussion

Among over ten thousand older adults in our study, the average PM_2.5_ and peak season O_3_ levels did not meet the WHO guideline interim target one. In contrast, the annual average NO_2_ has met the AQG level since 2015. The study found PM_2.5_ was the dominant mortality risk factor among the three pollutants. Interestingly, the oldest-old were less vulnerable to PM_2.5_ and NO_2_ than those younger than 80. The associations between air pollutants and mortality also varied in different districts of China and were influenced by temperature, road density, GDP per capita, and population density.

China has made policy efforts to reduce air pollution over the past decades, including the issuance of the Atmosphere Ten Articles in 2013 and the Blue Sky Defense War in 2018. These efforts have led to significant improvements in China’s air quality, with the ambient exposure for our study participants decreasing greatly from 59.6 to 39.4 μg/m^3^ during 2013–2019. According to the WHO, the data reporter of SDG target 11.6.2, the annual mean levels (μg/m^3^) of PM_2.5_ (95% CI) in China from 2010 to 2019 decreased from 47.18 (44.59–49.38) to 38.15 (36.69–39.42) in the total areas, 49.53 (46.83–51.71) to 40.17 (38.57–41.56) in urban areas, and 39.34 (37.15–41.47) to 31.13 (29.81–32.34) in rural areas.^5^ However, despite the decrease, the average levels of PM_2.5_ in the sampled areas in 2019 were still higher than the WHO interim target one of 35 μg/m^3^. The majority of places were also above the WHO AQG second interim target of 25 μg/m^3^.

Unlike developed countries, the change of NO_2_ in our study was relatively small, and the annual average level was lower than the WHO AQG level of 10 μg/m^3^ since 2014. NO_2_ increased from 2006 to 2011 and decreased since 2011. The change trend is similar to the findings of another study, which estimated the annual mean increase rate as 0.348 ± 0.132 μg/m^3^ between 2005 to 2011, and an annual mean rate of decrease as 0.312 ± 0.188 μg/m^3^ between 2012 and 2019.^24^

O_3_ has increased while PM_2.5_ decreased. O_3_ is formed through complex radical chain-reaction chemistry. There was a potential trade-off between O_3_ precursors emissions-reducing and particle pollution-reducing.^25^ The majority areas in mainland China also suffered peak season ozone concentrations that were higher than the first interim target: 100 μg/m^3^ of WHO 2021 guideline (target 2:70; AQG: 60 μg/m^3^).^26^ Peak season average O_3_ remained around 108 μg/m^3^ before 2015 and increased to around 123 μg/m^3^ in 2017 in our study. According to another study, the population-weighted median concentrations of predicted MDA8 ozone was 89.34 μg/m^3^ in mainland China in 2013 and reached 100.96 μg/m^3^ in 2019.^26^ The gap can be due to the difference in the sampling district, O_3_ source and estimation methods. There is no long-term annual limit for O_3_ in the Environment Air Quality Standard (GB3095-2012) of China so far. NO_2_ and O_3_ have not been included in the WHO SDG related indicator report yet.

The range of the last year PM_2.5_ was 14.8–133.0 μg/m^3^, with the lowest level higher than the WHO AQG target. The concentration–response curve showed no safe threshold for increased mortality risk. The steep relationship within about 60 μg/m^3^ indicated a stronger association in the relatively low exposure range than in the high range exposure. The last year’s annual average NO_2_ ranged from 1.2 to 109.0 μg/m^3^, with the lowest level lower than the WHO AQG level. The mortality risk also increased with the NO_2_ concentration increasing, and there was also no threshold. This should be new evidence for the guideline update. Notably, the source of NO_2_ was mainly the traffic. Road density was positively associated with NO_2_ and also negatively associated with mortality [HR (95% CI) of the fourth quartile vs first quartile: 0.72 (0.66, 0.78)]. Road density, GDP per capita, and population density were neglected confounders in most previous NO_2_ studies. Peak season O_3_ ranged from 20 to 182 μg/m^3^, with the lowest level lower than the WHO AQG level. Higher O_3_ was associated with lower mortality risk on average [HR (95% CI): 0.87 (0.86, 0.89) for annual O_3_ and 0.89 (0.87, 0.90) for peak season O_3_]. Previous evidence on long-term O_3_ and mortality risk is modest. Pooled results based on nine studies showed no significant association between increased all-year O_3_ exposure and all-cause mortality as HR being 0.97 (95% CI: 0.93, 1.02) per 10 μg/m^3^ with a large heterogeneity.^27^ A more recent cohort based meta-analysis found a positive association between long-term O_3_ and all-cause mortality [HR(95% CI): 1.014, 1.009–1.019 for 10 nmol mol^–1^ incremental warm season O_3_].^28^ However, this meta-analysis used large-scale O_3_ exposure health risk studies up to 2022, which did not cover Asian, African, or Latin American regions. The latest finding based on another Chinese cohort identified an HR of 1.18 (95% CI: 1.13, 1.23) per 10 μg/m^3^ increase of O_3_ and all-cause mortality.^29^ Their population had a mean age of 57.7 (SD: 10.4), while our study population was aged 80 on average. The different sources and resolution of O_3_ exposure could be other reasons for the contradictory findings. Besides, higher O_3_ was associated with higher mortality risk in the low range of about less than 110 μg/m^3^, and then the HR decreased to less than one in the high range of O_3_. Among those who were exposed to high ozone concentrations (exceeding 100 μg/m^3^), a substantial majority resided in eastern China, which is the most developed district of China. We suspect that there may be residual social economic confounding. Furthermore, the associations between NO_2_ and mortality became gradually weaker for longer exposure time windows of the last two or three years, while it was stable for PM_2.5_ and O_3_ (Table S3). More discussions on the separate association between air pollutants and mortality are in the extended discussion (see Extended discussion in Supporting Information).

O_3_ and secondary PM_2.5_ are both pollutants produced through chemical chain reactions of volatile organic compounds (VOCs) and oxides of nitrogen (NOx). Two-pollutant models can be difficult to interpret when the correlation between pollutants is high or exposures for pollutants are assessed with different methods or at a different spatial resolution.^2^ In the review, overall RRs for PM_2.5_ were much lower in studies that specified two pollutant models adjusted for NO_2_ compared to the single pollutant estimates [1.02 (95% CI 1.00, 1.04) in two-pollutant models versus 1.07 (95% CI 1.05, 1.08) in single pollutant models]. RRs remained stable after adjusting for O_3_: 1.08 (95% CI 1.04, 1.11) based upon seven studies, respectively.^2^ Associations between NO_2_ or O_3_ and mortality were attenuated upon adjustment for copollutants in some studies but not in others.^3^ In our study, the HR of PM_2.5_ increased after adjusting for peak season O_3_, and the HR of NO_2_ was attenuated significantly after adjusting for PM_2.5_, and the HR of O_3_ did not change much after adjusting for PM_2.5_ or NO_2_. In our previous study, the association between NO_2_ and mortality even became insignificant after adjusting for PM_2.5_ without adjustment of road density.^10^ The different sources of our PM_2.5_, NO_2_, and O_3_ data can limit the comparability. The correlations were not very strong, with the highest Pearson coefficient as 0.37 between PM_2.5_ and NO_2_. We also noticed that in another national study in China, the HR for mortality of NO_2_ even doubled after adjusting for PM_2.5._^30^ As discussed above, the population age, air pollution source, and resolution may lead to the inconsistency. PM_2.5_ was still the dominant pollutant associated with mortality in our study. A study using U.S. Medicare data also indicates that PM_2.5_ posed the greatest risk among PM_2.5_, NO_2_, and O_3._^9^ The LIFEWORK study in The Netherlands found PM_2.5_ was the most relevant contributor to the positive association with the mortality among a mixture of five components: PM_2.5_, NO_2_, etc.^31^

We also saw air pollution mortality risk was modified by individual level characteristics. It is thought there was a higher risk for individuals living in a lower socioeconomic status (SES). Those with higher SES tended to have more advantages in life habits and resources, health care access, and air pollution exposure reduction. In our study, relatively younger people, those living with a spouse or without cognitive impairment or ADL disability, were surprisingly more susceptible to PM_2.5_ and NO_2_. This may be related to more exposure to ambient air pollution through more outdoor activities for those younger and healthier participants. Another possible reason may be that other health issues afflicting the elderly population masked the effects of air pollutants. A Hong Kong study also found a higher risk for the younger group and stated this might be explained by the healthy survivor effect^32^ that those who survived are more resistant. The concentration–response relationship was also reported to be different in different ages. A study in Norway found that the risk of death of NO_2_ from all-causes started to increase from 40 μg/m^3^ in younger subjects, whereas the relationship was linear across the concentration range (2–73 μg/m^3^) in the oldest age group.^33^ Males had a nonsignificant higher mortality risk of both PM_2.5_ and NO_2_ than females, which was consistent with a previous study in Hong Kong,^32^ while some studies in the USA reported a significant gender difference.^34,35^ WHO reported age-standardized mortality rate (95% CI) attributed to ambient air pollution in China (per 100 000 population) was higher in males at 82.69 (64.83–102.8) compared to females at 48.41 (37.58–60.46).^5^ The mortality risk of those having a higher household income was impacted less by the air pollution in our study. But we also found that those with higher education did not have a lower risk for either PM_2.5_ or NO_2_. The stratified HRs for PM_2.5_ had a nonlinear trend with education level increasing and indicated weaker associations in the highest and lowest education group and stronger associations among the middle-level education group. We further found that this PM_2.5_ risk difference for the two education groups only existed in females, and not in males. People with less education in the US were at higher risk of mortality associated with air pollution exposure.^36^ The associations did not differ consistently by educational level in three Latin American cities.^37^ Another inconsistency was that there were different demographic and SES indexes in different countries. For example, there was a great difference in the air pollution risk among different races^34^ in the US population, while race may not be a significant factor in other countries. For different lifestyles, heavy smokers suffered greater from both PM_2.5_ and NO_2_, and heavy drinkers suffered greater from PM_2.5_ in our study.

The areal-level SES can interact with individual SES and play a role in the association with health. The effect size of PM_2.5_ for mortality was slightly larger in rural areas than in urban areas and tended to be higher in areas with lower road density and low population density. In China, a developing country, areas with higher road density and urban areas can have higher air pollution levels but also usually have richer social resources and higher economic levels. On the one hand, residents living in such areas can have a higher awareness of air pollution and adopt more methods to reduce personal exposure to air pollution. On the other hand, rich social resources like good health care in places with convenient transportation could reduce the mortality of the residents.

The effect size of PM_2.5_ for mortality was larger in the South than in the North of China despite PM_2.5_ being higher in the north than the south in our study, which was consistent with another study that used the same cohort for the Chinese old population aged 65 or older.^7^ O_3_ was also only harmful in southern areas in our study. The different geographic characteristics, climates, and lifestyles between northern and southern China can play a role. When stratified by temperature, PM_2.5_ and O_3_ both showed higher risk in areas with higher temperature and lower temperature variations. The frequency of opening windows in southern areas was also usually higher than in the north in winter, which can impact indoor air pollution exposure, especially for the O_3_, whose concentration was very different between indoors and outdoors. Another study for Chinese men aged 40 or older found a higher risk for the north than the south.^38^ They sampled different provinces, included only males, and surveyed a younger population compared to our study design.

Individual-level, area-level SES, and air pollution are intertwined.^39^ A multilevel cohort study in Norway found that the effect of social deprivation at the neighborhood level was independent of and stronger than the individual social deprivation in the association between air pollution and mortality.^40^ We also found that the geographic region affected the effect of PM_2.5_ and O_3_, and the road density, GDP, population density, and temperature affected the effect of PM_2.5_ and NO_2_ greatly. The residual area-level confounding may help explain the mixed results for the stratified analysis. These differences need further validation and call for more sensitivity analysis for subpopulations and specific contexts. It also indicated a need for specialized standards in addition to the overall target.

Our study has several strengths. We used a large cohort of oldest-old individuals, and followed up for 10 years, across multiple geographical areas. We accounted for multiple air pollutants over a long period of time. We adjusted for a variety of possible individual and area-level confounders such as age, sex, individual SES, individual smoking, other individual lifestyles, and area-level environment and economic confounders like road density, GDP per capita, climate region, and temperature.

However, there are also some limitations to our study. First, we used ambient air pollution derived from satellite data, which may not accurately represent real exposure. Second, different data sources and estimation methods were used for the air pollutants, which may limit comparability in the multipollutant model. Third, we used all-cause mortality, which limited our ability to test cause-specific mortality. Also, we did not consider comorbidities which can be a source of confounding. Lastly, our sample was mainly composed of participants aged 80 or older, and the majority lived in rural areas, which may limit the generalizability of our findings.

## Conclusions

Our study provides real-world evidence comparing to WHO AQG that contributes to a better understanding and addressing the indicators outlined in SDG 3.9 and 11.6, specifically focusing on advanced-aged adults in specific populations and regions. We observed a significant gap between current levels of PM_2.5_, O_3_ and the World Health Organization’s Air Quality Guideline (AQG) level, highlighting the need for continued efforts to reduce PM_2.5_ exposure. Additionally, despite the increase in road density, it is crucial to prioritize measures aimed at maintaining low levels of NO_2_. Further research is necessary to fully comprehend the relationship between individual and area-level SES and the risk posed by air pollution.

## Data Availability

The CLHLS data sets are available from the Peking University Open Research Data (http://opendata.pku.edu.cn/dataverse/CHADS) and Interuniversity Consortium at University of Michigan (https://www.icpsr.umich.edu/icpsrweb/NACDA/series/487).

## Supporting Information Available

(a) Supplementary methods: Study sample; Other Covariates and Modifiers Measurements; (b) Supplementary results: (b) Table S1. The characteristics and exposures across different age groups; Table S2. Air pollution across different groups; Figure S1. The Directed Acyclic Graph (DAG) of the relationship between ambient air pollution and mortality; Table S3 Association between the two-year and three-year air pollutants’ level and mortality risk; Figure S2. Association between air pollutants and mortality stratified by individual characteristics; (c) Extended discussion: Separate association between air pollutants and mortality (PDF)
