# Supplementary material for: Real-World Evidence of Multiple Air Pollutants and Mortality: A Prospective Cohort Study in an Oldest-Old Population
Source: Environ Health (Wash). 2023 Nov 9;2(1):23–33. doi: 10.1021/envhealth.3c00106 (PMC10804360; doi:10.1021/envhealth.3c00106)
Supplement: Supplementary file 1 — eh3c00106_si_001.pdf [file eh3c00106_si_001.pdf]

Supporting Information

Manuscript title: Real-World Evidence of Multiple Air Pollutants and Mortality: A Prospective Cohort Study in An Oldest-Old Population

Authors: Linxin Liu[1,2], Yi Zeng[3,4], John S. Ji[1*]

Author affiliations:

1. Vanke School of Public Health, Tsinghua University, Beijing, China, 100084
2. School of Medicine, Tsinghua University, Beijing, China, 100084
3. Center for the Study of Aging and Human Development, School of Medicine, Duke University, Durham, NC, USA, 27710
4. Center for Healthy Aging and Development Studies, National School of Development, Peking University, Beijing, China, 100091

*Corresponding to John S. Ji, johnji@tsinghua.edu.cn

Supplementary methods

Study Sample

Among the 16 954 participants in the 2008/2009 cohort, we excluded 391 participants aged younger than 65, 2856 participants who were lost in the first follow-up or did not have death time record, 268 participants without matched NO_2 or $PM_{2.5}$, and 232 participants with missing values in covariates. For the household income variable with more than 1000 missing values, a separate group was coded as "missing". We finally included 13,207 participants.

Other Covariates and Modifiers Measurements

The CLHLS asks for date of birth (rather than age directly) and computes the respondent's age after the survey by subtracting it from the date of the survey and the age was validated by other information including genealogical record, ID card, and household registration booklet. We classified marital status into two categories: Currently married and living with spouse as "married" and widowed/separated/divorced/Never married/married but not living with spouse as "not married." We used the schooling year to evaluate education level and further classified the schooling year into three groups: 0 years (without formal education), 1-6 years (with primary education), and >6 years (with higher education). We divided the regular exercise into three categories: "Current", "Former", and "Never". We classified smoking and alcohol-drinking status into four categories: "Heavy smoker" or "Heavy drinker", "Moderate smoker" or "Moderate drinker", "Former", and "Never". For example, participants were asked, "do you do exercise regularly at present (planned exercise like walking, playing balls, running, and so on)?" and/or "did you do exercise regularly in the past?". We defined the regular exercise status as "Current" for participants who answered "Yes" to the first question, "Former" for those who answered "No" to the first question and "Yes" to the second question, and "Never" for who answered "No" to both two questions. Then we further quantified the current smoker based on the number of times they smoke (or smoked) per day: <20 times/day and ≥20 times/day. We also quantified the current alcohol drinker based on the kind of alcohol and how much they drank daily. The unit of alcohol was a Chinese unit of weight called 'Liang' [50 grams (g)]. The level of alcohol consumption was calculated as drinks of alcohol per day, based on the beverage type and amount, assuming the following alcohol content by volume (v/v) typically seen in China: strong liquor 53%, weak liquor 38%, grape wine 12%, rice wine 15%, and beer 4% [1]. A standard drink was equal to 14.0 g of pure alcohol according to the criterion of the Center for Disease Control and Prevention in the USA, and moderate drinking is up to 1 drink per day for women and up to 2 drinks per day for men according to Dietary Guidelines for Americans 2015-2020. Therefore, we defined those who drank equal to or less than 14 g pure alcohol per day for the female or 28 g per day for the male as light drinkers, otherwise heavy drinkers. The participant was asked directly how much the household income is. There were four categories for household income (yuan): <4000, <10000, <20000, and ≥20000. Co-residence was classified according to the answer to the question "who do you live with?": living with the family, alone, or nursing center. The frequency of housework and social activity were classified as every day, occasionally, and never according to the questionnaire.

We calculated BMI as body weight divided by the square of the body height (unit: kg/m^2). We used the WHO standard of BMI, which defined a BMI of <18.5 kg/m^2 as underweight, a BMI of ≥ 18.5 to <25 kg/m^2 as normal weight, a BMI of ≥ 25 as overweight/obese. We used an adapted Chinese version of the MMSE to assess the cognitive function of the participants. The scale is 0–30 points, with a higher

score indicating better cognitive function. We classified it into three groups: ≥24 (normal cognition), 18–23 (mild cognitive impairment), and <18 (serious cognitive impairment). For ADL, the participant scored one for the respondent who needed help in performing any of six daily activities (bathing, dressing, eating, indoor movement, toileting, and continence) and the total score was classified as 0 score, 1 score, and >1 score.

Supplementary results

Table S1. The characteristics and exposures across different age groups

Age group	<80 (N=3527)	80-89 (N=3445)	90-99 (N=3627)	100~ (N=2608)	Overall (N=13207)
**Age**					
Mean (SD)	72.1 (4.19)	84.7 (2.99)	93.5 (2.73)	102 (2.35)	87.2 (11.4)
Median [Min, Max]	72.0 [65.0, 79.0]	85.0 [80.0, 89.0]	93.0 [90.0, 99.0]	102 [100, 116]	89.0 [65.0, 116]
**Gender**					
Male	1857 (52.7%)	1733 (50.3%)	1494 (41.2%)	489 (18.8%)	5573 (42.2%)
Female	1670 (47.3%)	1712 (49.7%)	2133 (58.8%)	2119 (81.3%)	7634 (57.8%)
**Education**					
0 year	1461 (41.4%)	2153 (62.5%)	2592 (71.5%)	2258 (86.6%)	8464 (64.1%)
1-6 years	1462 (41.5%)	987 (28.7%)	806 (22.2%)	282 (10.8%)	3537 (26.8%)
>6 years	604 (17.1%)	305 (8.9%)	229 (6.3%)	68 (2.6%)	1206 (9.1%)
**Marriage**					
Married	2305 (65.4%)	1174 (34.1%)	480 (13.2%)	85 (3.3%)	4044 (30.6%)
not married	1222 (34.6%)	2271 (65.9%)	3147 (86.8%)	2523 (96.7%)	9163 (69.4%)
**Regular exercise**					
Current	1375 (39.0%)	962 (27.9%)	788 (21.7%)	404 (15.5%)	3529 (26.7%)
Former	328 (9.3%)	388 (11.3%)	559 (15.4%)	366 (14.0%)	1641 (12.4%)
Never	1824 (51.7%)	2095 (60.8%)	2280 (62.9%)	1838 (70.5%)	8037 (60.9%)
**Smoking**					
Never	1992 (56.5%)	2125 (61.7%)	2543 (70.1%)	2149 (82.4%)	8809 (66.7%)
Former	609 (17.3%)	644 (18.7%)	565 (15.6%)	270 (10.4%)	2088 (15.8%)
Light smoker	637 (18.1%)	566 (16.4%)	444 (12.2%)	170 (6.5%)	1817 (13.8%)

Age group	<80 (N=3527)	80-89 (N=3445)	90-99 (N=3627)	100~ (N=2608)	Overall (N=13207)
Heavy smoker	289 (8.2%)	110 (3.2%)	75 (2.1%)	19 (0.7%)	493 (3.7%)
**Drinking**					
Never	2238 (63.5%)	2277 (66.1%)	2599 (71.7%)	2024 (77.6%)	9138 (69.2%)
Former	530 (15.0%)	550 (16.0%)	508 (14.0%)	262 (10.0%)	1850 (14.0%)
Moderate drinker	239 (6.8%)	270 (7.8%)	219 (6.0%)	107 (4.1%)	835 (6.3%)
Heavy drinker	520 (14.7%)	348 (10.1%)	301 (8.3%)	215 (8.2%)	1384 (10.5%)
**Household income**					
<4000	793 (22.5%)	828 (24.0%)	767 (21.1%)	425 (16.3%)	2813 (21.3%)
<10000	799 (22.7%)	652 (18.9%)	720 (19.9%)	587 (22.5%)	2758 (20.9%)
<20000	741 (21.0%)	694 (20.1%)	720 (19.9%)	550 (21.1%)	2705 (20.5%)
>=20000	970 (27.5%)	927 (26.9%)	1088 (30.0%)	864 (33.1%)	3849 (29.1%)
Missing	224 (6.4%)	344 (10.0%)	332 (9.2%)	182 (7.0%)	1082 (8.2%)
**Residence**					
Urban	1313 (37.2%)	1222 (35.5%)	1365 (37.6%)	867 (33.2%)	4767 (36.1%)
Rural	2214 (62.8%)	2223 (64.5%)	2262 (62.4%)	1741 (66.8%)	8440 (63.9%)
**Region**					
Central	790 (22.4%)	805 (23.4%)	843 (23.2%)	618 (23.7%)	3056 (23.1%)
Eastern	1179 (33.4%)	1037 (30.1%)	1101 (30.4%)	741 (28.4%)	4058 (30.7%)
Northeastern	290 (8.2%)	196 (5.7%)	203 (5.6%)	249 (9.5%)	938 (7.1%)
Northern	215 (6.1%)	206 (6.0%)	191 (5.3%)	93 (3.6%)	705 (5.3%)
Southern	700 (19.8%)	703 (20.4%)	803 (22.1%)	504 (19.3%)	2710 (20.5%)
Southwestern	353 (10.0%)	498 (14.5%)	486 (13.4%)	403 (15.5%)	1740 (13.2%)

Age group	<80 (N=3527)	80-89 (N=3445)	90-99 (N=3627)	100~ (N=2608)	Overall (N=13207)
**Road density (meters/5km radius)**					
Mean (SD)	1420 (1310)	1290 (1210)	1300 (1250)	1190 (1250)	1310 (1260)
Median [Min, Max]	1050 [0, 7170]	915 [0, 7230]	913 [0, 7290]	735 [0, 7160]	896 [0, 7290]
**Temperature annual mean**					
Mean (SD)	16.4 (4.11)	16.6 (3.85)	16.6 (3.98)	15.9 (4.21)	16.4 (4.04)
Median [Min, Max]	16.7 [0.902, 25.3]	16.8 [0.902, 25.2]	16.7 [0.132, 25.1]	16.0 [2.42, 25.3]	16.7 [0.132, 25.3]
**Temperature annual SD**					
Mean (SD)	9.18 (2.14)	8.94 (1.97)	8.95 (2.01)	9.27 (2.16)	9.07 (2.07)
Median [Min, Max]	9.24 [4.28, 16.9]	9.04 [4.28, 17.2]	9.15 [4.28, 17.1]	9.42 [4.28, 17.1]	9.21 [4.28, 17.2]
**Elevation (meter)**					
Mean (SD)	139 (188)	158 (201)	146 (199)	155 (191)	149 (195)
Median [Min, Max]	52.0 [-2.00, 1360]	66.0 [-2.00, 1390]	58.0 [-2.00, 2270]	64.0 [0, 2110]	59.0 [-2.00, 2270]
**GDP per capita in 2008 (yuan)**					
Mean (SD)	30300 (18700)	28800 (18000)	28300 (17700)	27500 (17900)	28800 (18100)
Median [Min, Max]	22700 [6480, 79800]	20900 [6480, 79800]	20900 [6480, 79800]	20900 [6480, 80700]	21000 [6480, 80700]
**Population density in 2008 (per square kilometer)**					
Mean (SD)	618 (495)	594 (413)	624 (495)	638 (546)	617 (486)

Age group	<80 (N=3527)	80-89 (N=3445)	90-99 (N=3627)	100~ (N=2608)	Overall (N=13207)
Median [Min, Max]	522 [25.3, 3380]	519 [25.3, 3380]	529 [25.3, 3380]	546 [39.0, 3380]	529 [25.3, 3380]
**Coresidence status**					
Colive	3026 (85.8%)	2744 (79.7%)	2916 (80.4%)	2335 (89.5%)	11021 (83.4%)
Alone	478 (13.6%)	645 (18.7%)	643 (17.7%)	213 (8.2%)	1979 (15.0%)
Institution	23 (0.7%)	56 (1.6%)	68 (1.9%)	60 (2.3%)	207 (1.6%)
**Number of coresidence people**					
[0, 2)	1969 (55.8%)	1691 (49.1%)	1304 (36.0%)	633 (24.3%)	5597 (42.4%)
2	419 (11.9%)	522 (15.2%)	880 (24.3%)	976 (37.4%)	2797 (21.2%)
3	397 (11.3%)	400 (11.6%)	438 (12.1%)	295 (11.3%)	1530 (11.6%)
[4,29]	742 (21.0%)	832 (24.2%)	1005 (27.7%)	704 (27.0%)	3283 (24.9%)
**Housework frequency**					
everyday	2421 (68.6%)	1603 (46.5%)	837 (23.1%)	286 (11.0%)	5147 (39.0%)
occasionally	531 (15.1%)	540 (15.7%)	496 (13.7%)	240 (9.2%)	1807 (13.7%)
never	575 (16.3%)	1302 (37.8%)	2294 (63.2%)	2082 (79.8%)	6253 (47.3%)
**Social activity frequency**					
everyday	169 (4.8%)	72 (2.1%)	51 (1.4%)	16 (0.6%)	308 (2.3%)
occasionally	565 (16.0%)	294 (8.5%)	202 (5.6%)	54 (2.1%)	1115 (8.4%)
never	2793 (79.2%)	3079 (89.4%)	3374 (93.0%)	2538 (97.3%)	11784 (89.2%)
**BMI group**					
<18.5	648 (18.4%)	1059 (30.7%)	1437 (39.6%)	1123 (43.1%)	4267 (32.3%)
[18.5,25)	2332 (66.1%)	2077 (60.3%)	1868 (51.5%)	1188 (45.6%)	7465 (56.5%)

Age group	<80 (N=3527)	80-89 (N=3445)	90-99 (N=3627)	100~ (N=2608)	Overall (N=13207)
>=25	528 (15.0%)	257 (7.5%)	205 (5.7%)	131 (5.0%)	1121 (8.5%)
Missing	19 (0.5%)	52 (1.5%)	117 (3.2%)	166 (6.4%)	354 (2.7%)
**MMSE score**					
<18	118 (3.3%)	558 (16.2%)	1383 (38.1%)	1709 (65.5%)	3768 (28.5%)
[18,24)	279 (7.9%)	681 (19.8%)	831 (22.9%)	397 (15.2%)	2188 (16.6%)
>=24	3130 (88.7%)	2206 (64.0%)	1413 (39.0%)	502 (19.2%)	7251 (54.9%)
**ADL score**					
0 score	3400 (96.4%)	3032 (88.0%)	2669 (73.6%)	1294 (49.6%)	10395 (78.7%)
1 score	57 (1.6%)	193 (5.6%)	361 (10.0%)	435 (16.7%)	1046 (7.9%)
>1 score	70 (2.0%)	220 (6.4%)	597 (16.5%)	879 (33.7%)	1766 (13.4%)
**Annual $PM_{2.5}$ (μg/m^3)**					
Mean (SD)	48.4 (15.3)	52.5 (15.8)	54.4 (15.4)	56.7 (15.5)	52.8 (15.8)
Median [Min, Max]	45.9 [14.8, 128]	51.1 [17.9, 131]	52.4 [15.4, 132]	55.1 [19.5, 133]	51.2 [14.8, 133]
**Annual NO_2 (μg/m^3)**					
Mean (SD)	18.3 (13.5)	18.1 (12.0)	19.6 (14.1)	20.3 (15.4)	19.0 (13.8)
Median [Min, Max]	15.0 [1.24, 109]	15.9 [1.34, 102]	16.8 [1.27, 102]	17.2 [1.22, 107]	16.2 [1.22, 109]
**Peak season O_3 (μg/m^3)**					
Mean (SD)	114 (22.7)	110 (20.9)	108 (20.2)	109 (20.2)	110 (21.2)
Median [Min, Max]	115 [45.2, 172]	112 [23.7, 182]	110 [20.3, 170]	111 [42.2, 168]	112 [20.3, 182]
**Annual O_3 (μg/m^3)**					
Mean (SD)	94.4 (13.0)	91.6 (11.8)	91.0 (11.0)	90.5 (11.0)	92.0 (11.9)
Median [Min, Max]	94.3 [45.7, 147]	91.7 [34.1, 127]	91.1 [21.4, 144]	90.4 [48.9, 143]	91.9 [21.4, 147]

Table S2. Air pollution across different groups

	Overall (N=13207)	Annual $PM_{2.5}$ ($\mu g/m^3$)		Annual NO_2 ($\mu g/m^3$)		Annual O_3 ($\mu g/m^3$)		Peak season O_3 ($\mu g/m^3$)	
		Mean (SD)	Median (P25-P75)	Mean (SD)	Median (P25-P75)	Mean (SD)	Median (P25-P75)	Mean (SD)	Median (P25-P75)
**Overall**		52.8 (15.8)	51.2 [41.2, 63]	19.0 (13.8)	16.2 [10.5, 23.1]	92.0 (11.9)	91.9 (83.7, 100.3)	110 (21.2)	112 [94.4, 124.9]
**Age group**									
<80	3527 (26.7%)	48.4 (15.3)	45.9 (37.1-58.7)	18.3 (13.5)	15 (9.9-22.6)	94.4 (13)	94.3 (84.8-104.2)	114 (22.7)	115.1 (96.7-128.5)
80-89	3445 (26.1%)	52.5 (15.8)	51.1 (41.1-62.4)	18.1 (12)	15.9 (10.3-22.6)	91.6 (11.8)	91.7 (83.8-100)	110.1 (20.9)	112 (94.6-123.7)
90-99	3627 (27.5%)	54.4 (15.4)	52.4 (43.6-64.2)	19.6 (14.1)	16.8 (11-23.5)	91 (11)	91.1 (83.6-98.7)	108.2 (20.2)	110 (92.6-123.4)
100~	2608 (19.7%)	56.7 (15.5)	55 (45.9-67.4)	20.3 (15.4)	17.2 (10.9-24.1)	90.5 (11)	90.4 (82.9-98.3)	109.1 (20.2)	111.4 (94-124.1)
**Gender**									
Male	5573 (42.2%)	52.2 (15.8)	50.6 (40.6-62.6)	19.2 (13.9)	16.4 (10.6-23.4)	92.2 (12)	92.2 (83.8-100.6)	110.6 (21.5)	112.4 (94.5-125)
Female	7634 (57.8%)	53.2 (15.7)	51.6 (41.8-63.4)	18.9 (13.6)	16.2 (10.4-22.9)	91.8 (11.8)	91.7 (83.7-100.1)	110.3 (21)	112 (94.3-124.8)
**Education**									
0 year	8464 (64.1%)	53.7 (15.5)	52.5 (42.5-64.2)	18 (12.2)	16 (10.3-22.2)	92 (11.6)	92 (83.9-100.1)	110.4 (20.7)	112.3 (95.1-124.8)
1-6 years	3537 (26.8%)	50.4 (15.5)	48.2 (39.3-58.9)	19.6 (15.1)	15.9 (10.3-24.2)	92 (12.3)	91.8 (83.5-100.8)	109.9 (21.9)	111.5 (92.6-125)
>6 years	1206 (9.1%)	52.7 (17.4)	50.3 (40.4-62.8)	24.5 (17.8)	19.7 (12.1-32.8)	92 (12.5)	91.9 (83-101)	112 (22.5)	113.6 (94.7-125.8)
**Marriage**									
Married	4044 (30.6%)	50 (15.6)	48.3 (38.5-59.9)	19 (13.4)	16.1 (10.7-23.4)	93.7 (12.5)	93.5 (84.8-102.3)	112.9 (22.1)	115.1 (96.7-127.2)
not married	9163 (69.4%)	54 (15.7)	52.3 (42.8-64.2)	19 (13.9)	16.3 (10.3-23)	91.2 (11.5)	91.1 (83.3-99.3)	109.3 (20.7)	111.1 (93.5-124)
**Co-residence status**									
live with the family	11021 (83.4%)	53.3 (15.9)	51.7 (41.7-63.6)	19.3 (13.9)	16.5 (10.7-23.6)	92 (11.9)	92 (83.7-100.3)	110.9 (21.1)	112.7 (94.9-125.3)
alone	1979 (15.0%)	49.9 (14.7)	48.1 (39.4-59.6)	17 (12.5)	14.6 (9.4-21.2)	92.1 (11.6)	91.7 (83.7-100.2)	108.2 (21.9)	109.4 (92.6-122.7)
institution	207 (1.6%)	50.2 (13.8)	49 (40.6-59.1)	22.4 (15.4)	18.4 (12.7-28.1)	91.9 (11.1)	92.1 (84.5-99.7)	106.8 (17.3)	105.6 (93.2-118.9)

	Overall (N=13207)	Annual $PM_{2.5}$ (μg/m³)		Annual NO_2 (μg/m³)		Annual O_3 (μg/m³)		Peak season O_3 (μg/m³)	
		Mean (SD)	Median (P25-P75)	Mean (SD)	Median (P25-P75)	Mean (SD)	Median (P25-P75)	Mean (SD)	Median (P25-P75)
**Regular exercise**									
Current	3529 (26.7%)	52 (16.4)	50.5 (40.3-62.3)	21.7 (15.3)	18 (12-27.2)	91.7 (12.7)	91.3 (83.4-100.9)	111.4 (22.5)	112.6 (94.7-126.1)
Former	1641 (12.4%)	54.1 (17.8)	52 (42.2-64.2)	21.9 (16.5)	18 (11-27.7)	90.4 (11.6)	90.7 (82.2-98.8)	109.1 (22)	111.2 (94.2-124.5)
Never	8037 (60.9%)	52.8 (15)	51.3 (41.5-63.2)	17.2 (12)	15.2 (9.8-21.4)	92.4 (11.6)	92.4 (84.2-100.4)	110.3 (20.4)	112.2 (94.3-124.5)
**Smoking**									
Never	8809 (66.7%)	52.5 (16.1)	50.8 (40.9-62.9)	18.7 (14)	15.9 (10-22.8)	91.6 (11.8)	91.4 (83.5-100)	109.4 (21.4)	111.1 (92.6-124.6)
Former	2088 (15.8%)	54.2 (15.8)	52.5 (42.9-64.3)	20.9 (13.7)	18 (12.2-26)	92.3 (11.8)	92.4 (83.9-100.8)	112 (20.5)	113.6 (97.6-125.3)
Light smoker	1817 (13.8%)	53.1 (14)	51.8 (42.6-63)	18.7 (12.5)	16.4 (10.9-22.9)	93 (12.2)	93.2 (85.2-101)	113.2 (20)	114.9 (99.5-126)
Heavy smoker	493 (3.7%)	49.2 (14.9)	48.3 (38.5-58.3)	17 (12.5)	14.8 (9.4-20.9)	93.1 (12.7)	92.9 (84-101.9)	110.7 (22.7)	112.5 (93-125.9)
**Drinking**									
Never	9138 (69.2%)	52.9 (16.1)	51.1 (41.4-63.2)	19.2 (14.1)	16.3 (10.5-23.3)	92 (11.9)	91.8 (83.7-100.3)	110.2 (21.6)	112 (93.7-125.2)
Former	1850 (14.0%)	52.8 (15.3)	51.4 (41.3-63.2)	19.3 (13.4)	16.6 (10.9-24)	91.1 (11.8)	91.6 (83.1-99.1)	109.2 (20.2)	110.1 (94.3-122.4)
Moderate drinker	835 (6.3%)	53.5 (15.4)	52.1 (42.1-63.3)	19.5 (14.7)	15.9 (10.6-23.3)	91.9 (11.9)	92.1 (84.2-100.1)	111.8 (21.1)	113.5 (96.3-125.5)
Heavy drinker	1384 (10.5%)	51.5 (14.7)	50.5 (40.2-61.4)	17 (10.8)	15.3 (9.9-21.4)	93.3 (12.1)	93.2 (84.4-101.9)	112.7 (19.8)	114.5 (97.9-125.9)
**Housework frequency**									
everyday	5147 (39.0%)	49.1 (14.8)	47.5 (38.3-58.9)	17.2 (12.8)	14.5 (9.3-21.4)	92.6 (12.6)	92.2 (83.6-101.4)	110.3 (22.3)	111.5 (92.8-125)
occasionally	1807 (13.7%)	52.7 (16.4)	50.9 (40.1-63.5)	18.4 (13.4)	15.4 (9.6-23.3)	91.7 (11.8)	91.7 (83.6-100.2)	109.5 (20.8)	111.4 (93.1-124)
never	6253 (47.3%)	55.8 (15.8)	54 (44.6-66)	20.6 (14.4)	17.9 (12-24.6)	91.5 (11.3)	91.8 (83.9-99.5)	110.8 (20.4)	112.9 (96.3-125.1)

	Overall (N=13207)	Annual $PM_{2.5}$ (μg/m^3)		Annual NO_2 (μg/m^3)		Annual O_3 (μg/m^3)		Peak season O_3 (μg/m^3)	
		Mean (SD)	Median (P25-P75)	Mean (SD)	Median (P25-P75)	Mean (SD)	Median (P25-P75)	Mean (SD)	Median (P25-P75)
**Social activity frequency**									
everyday	308 (2.3%)	45.4 (16.8)	43.3 (33.4-53.2)	22.7 (15.6)	19.3 (12.3-29.2)	90.6 (13.8)	90 (80.3-101.3)	106.7 (24.4)	107 (88.9-122.8)
occasionally	1115 (8.4%)	49.8 (15.9)	47.6 (37.8-59.5)	21.7 (15)	18.2 (11.6-28)	93.3 (12.4)	93.2 (85-101.9)	111.3 (23.1)	112.7 (93.2-125.6)
never	11784 (89.2%)	53.2 (15.7)	51.6 (41.8-63.5)	18.6 (13.5)	16 (10.3-22.6)	91.9 (11.8)	91.8 (83.7-100.2)	110.4 (20.9)	112.2 (94.8-124.8)
**BMI group**									
<18.5	4267 (32.3%)	51.3 (15.4)	49.7 (40.3-61.9)	17 (13)	14.6 (8.7-21.3)	90.1 (11.3)	89.8 (82.6-97.7)	104.8 (21.5)	105.7 (87.5-121.2)
[18.5,25)	7465 (56.5%)	52.9 (15.7)	51.3 (41.2-63.2)	19.5 (14)	16.5 (10.7-23.8)	92.4 (12)	92.5 (84.1-100.8)	111.9 (20.3)	113.3 (97-125.2)
≥25	1121 (8.5%)	56.1 (17.4)	53.2 (43.6-66.4)	23.2 (14.8)	19.5 (13.8-27.9)	94.9 (12.9)	95.6 (85-103.8)	119.6 (21)	119.1 (106.3-133.8)
Missing	354 (2.7%)	55.6 (14.1)	53.3 (48-63.3)	19.8 (8.3)	18.4 (15-23.1)	95.7 (9.2)	96.7 (89.7-101.7)	118.5 (18.1)	121.1 (111.2-129.5)
**MMSE score**									
<18	3768 (28.5%)	56.7 (15)	55.5 (46.3-66.8)	19.8 (13.9)	17.4 (11.8-23.2)	91.5 (10.9)	92 (84-99.4)	111.1 (19.5)	113.7 (98-124.9)
[18,24)	2188 (16.6%)	52.9 (15.6)	51.1 (41.5-63.1)	18.2 (12.6)	16.1 (10.3-22.6)	91.9 (11.5)	92 (84.2-100.2)	109.8 (20.3)	112 (94.8-124.1)
≥24	7251 (54.9%)	50.7 (15.8)	48.7 (39.2-60.6)	18.8 (14)	15.4 (9.9-23.3)	92.2 (12.5)	91.8 (83.5-101)	110.3 (22.3)	111.4 (92.6-125.2)
**ADL score**									
0 score	10395 (78.7%)	51.4 (15.4)	49.9 (40.1-61.8)	17.7 (12.6)	15.2 (9.7-22.1)	92 (12.1)	91.8 (83.6-100.5)	109.7 (21.5)	111.1 (93.1-124.2)
1 score	1046 (7.9%)	58.6 (16.9)	55.6 (47.5-68.6)	25.1 (17)	20.5 (15-30.1)	92 (11)	92.5 (84.5-99.7)	115.6 (19.5)	117.4 (103.9-128.2)
>1 score	1766 (13.4%)	57.1 (15.6)	55.2 (46.1-67.4)	22.8 (16.2)	19 (13.2-27.5)	91.8 (10.9)	92.3 (84.2-99.7)	111.8 (19.6)	114.9 (98.4-125.7)
**Annual household income (yuan)**									

	Overall (N=13207)	Annual $PM_{2.5}$ (μg/m^3)		Annual NO_2 (μg/m^3)		Annual O_3 (μg/m^3)		Peak season O_3 (μg/m^3)	
		**Mean (SD)**	**Median (P25-P75)**	**Mean (SD)**	**Median (P25-P75)**	**Mean (SD)**	**Median (P25-P75)**	**Mean (SD)**	**Median (P25-P75)**
<4000	2813 (21.3%)	52.2 (15.8)	51.5 (40.1-64.1)	14.8 (8.7)	14 (9.2-19.3)	92.7 (11.9)	92.4 (84-100.8)	110.6 (22.5)	112.7 (94.2-126.2)
[4000,10000]	2758 (20.9%)	51.9 (15.1)	50.9 (40.1-62.8)	15.8 (9.2)	15 (9.4-20.3)	92 (11.6)	92 (83.8-100)	110.2 (20.4)	112.2 (95.4-123.9)
[10000,20000]	2705 (20.5%)	52.8 (15.2)	51 (41.8-62.3)	18.8 (12.6)	16.3 (10.7-23.3)	90.6 (12)	90.6 (82.4-98.9)	109 (20.3)	110.5 (93-123.5)
≥20000	3849 (29.1%)	54.2 (16.8)	51.6 (42.6-63.4)	24.7 (18.3)	19.9 (12.6-31.9)	92.3 (12)	92.2 (83.9-101)	111.7 (21.7)	113 (94.2-126.2)
Missing	1082 (8.2%)	51.2 (14.8)	49.8 (40.9-59.8)	18 (10.8)	16.9 (11-22.7)	92.4 (11.7)	92.4 (84.8-100.7)	109.4 (19.6)	111.7 (95.8-122.2)
**Residence**									
Urban	4767 (36.1%)	52.7 (16.2)	50.2 (41.9-61.4)	26.1 (18)	20.9 (13.9-33.9)	91.9 (12.2)	92.3 (83.5-101.2)	111.7 (20.2)	112.7 (97.2-125.2)
Rural	8440 (63.9%)	52.8 (15.5)	51.7 (40.9-63.7)	15 (8.2)	14.3 (9-19.9)	92 (11.7)	91.7 (83.9-100)	109.7 (21.7)	111.8 (92.7-124.8)
**Region**									
Central	3056 (23.1%)	61.3 (13.7)	62.4 (51.9-70.4)	16.2 (8.1)	15.1 (10.5-20.1)	92.5 (12.2)	92.2 (84.4-100.3)	114.2 (19)	114 (101.2-126.2)
Eastern	4058 (30.7%)	50.8 (12.6)	50.1 (41.8-58.1)	23 (15.6)	19.4 (14.5-25.8)	99.5 (9.3)	100 (93.2-105.5)	120.9 (15.9)	121.8 (111.2-131)
Northeastern	938 (7.1%)	47.8 (12.3)	47.9 (39.2-57.2)	27.1 (13.4)	25.1 (17.4-35)	85.1 (10.9)	85.2 (79.3-92.8)	106.1 (17.5)	106.7 (93.8-117.9)
Northern	705 (5.3%)	74.7 (22.2)	73.3 (57.2-94.2)	35 (18.2)	31 (19.9-50.5)	91.5 (11.9)	91.7 (82.6-100.2)	130.8 (19.4)	128.9 (117.2-146.5)
Southern	2710 (20.5%)	40.1 (9.4)	40.6 (33.9-47)	11.3 (9.2)	9.4 (4.3-14.7)	86.9 (9.5)	85.7 (80.2-92.4)	87.4 (16.2)	85.3 (78.8-93.5)
Southwestern	1740 (13.2%)	55.9 (12.8)	56.7 (45.3-66.4)	15.7 (10)	14.2 (8.7-19.9)	85.3 (10.1)	85.7 (78.2-92.8)	109.3 (13.2)	110.9 (100.8-118)
**Road density (meters/5km radius)**									
[0, 343)	3451 (26.1%)	53.3 (15.3)	53.1 (41.9-64.3)	12.3 (6.8)	11.5 (6.6-17.2)	91.6 (11.8)	91.2 (83.5-99.1)	108.6 (21.3)	110.4 (92.9-123.2)
[343, 992)	3450 (26.1%)	51.1 (15.5)	49.7 (39.9-61.4)	13.6 (7.5)	13.2 (8.1-18.3)	91.3 (11.8)	90.6 (83-99.4)	107.7 (21.8)	109.2 (90-123.6)
[992,2166)	3389 (25.7%)	51.4 (15)	49.9 (40.5-60)	19 (9)	17.7 (13.1-23.2)	92.6 (11.7)	92.4 (84.5-100.6)	110.6 (20.3)	112.4 (95.2-124.5)
[2166,7296]	2917 (22.1%)	55.6 (17.1)	52.1 (43.7-66)	33.3 (18.6)	28.6 (19.6-42)	92.4 (12.3)	92.9 (84-101.8)	115.7 (20.4)	116.7 (102.4-128.4)

	Overall (N=13207)	Annual $PM_{2.5}$ (μg/m³)		Annual NO_2 (μg/m³)		Annual O_3 (μg/m³)		Peak season O_3 (μg/m³)	
		Mean (SD)	Median (P25-P75)	Mean (SD)	Median (P25-P75)	Mean (SD)	Median (P25-P75)	Mean (SD)	Median (P25-P75)
**Temperature annual mean**									
[0.132,14.3)	3302 (25.0%)	56.8 (17.7)	53.1 (45.1-66.1)	25.3 (13.5)	21.5 (16.5-30.1)	93.1 (11.3)	94 (85.4-101.4)	120.3 (17.9)	120.7 (110-130.6)
[14.330,16.7)	3307 (25.0%)	60.8 (13.1)	61.3 (52.3-68.9)	20.7 (9.9)	18.7 (14.4-24.7)	97.2 (12.5)	97.1 (88.4-105.9)	122 (17.8)	121.8 (110.7-132.9)
[16.660,18.6)	3299 (25.0%)	52.8 (13)	51.7 (42.5-62.9)	18.2 (17.5)	13.3 (8.4-20.2)	90.4 (11.9)	91.4 (81.7-99.4)	109.7 (15.7)	110.8 (98-120.8)
[18.586,25.3]	3299 (25.0%)	40.6 (10.5)	40.3 (33.6-47)	11.7 (8.8)	10.3 (4.9-15.2)	87.2 (9.3)	86.6 (80.9-92.8)	89.7 (15.9)	87.3 (80.3-98.2)
**Temperature annual SD**									
[4.28, 7.59)	3308 (25.0%)	42.6 (12.4)	41.5 (34.3-49)	13.2 (10)	11.4 (5.2-17.1)	86.7 (9.8)	86 (79.9-92.6)	91.9 (17.5)	88.4 (80.6-104.6)
[7.59, 9.21)	3299 (25.0%)	51.1 (12.7)	49.9 (41.4-60.4)	15.9 (14.1)	12.5 (7.6-18.7)	89.8 (11.4)	90.1 (81.8-97.7)	106.4 (15.7)	105.6 (94.2-118.6)
[9.21,10.16)	3300 (25.0%)	58 (12.5)	58.9 (49.2-66.4)	21.3 (13.6)	18.3 (13.6-24.4)	97.5 (10.9)	98 (90.5-104.4)	120.3 (15.3)	120.6 (111-130)
[10.16,17.18]	3300 (25.0%)	59.3 (18.7)	56.3 (46.1-71.6)	25.7 (13.4)	21.8 (16.7-30.7)	93.9 (12.4)	94 (85.3-102.4)	123.2 (19.7)	122.9 (111.3-135.5)
**Elevation (meter)**									
[-2, 24)	3372 (25.5%)	49.2 (11.8)	48.6 (41.5-56.3)	25.5 (17)	21.3 (15.2-29.5)	98.1 (10.4)	99.4 (91.1-105.2)	115.9 (18.8)	119.1 (104.7-128)
[24, 60)	3263 (24.7%)	60.5 (16.4)	62 (50.4-70.8)	19.9 (12)	17.5 (12.7-22.5)	95 (11.5)	95.5 (87.4-102.4)	118.5 (21.7)	120.7 (107.8-131.9)
[60, 205)	3272 (24.8%)	50.5 (17.5)	47.3 (38.5-58.5)	15.9 (10.8)	13.2 (8.4-20.4)	88.8 (11.3)	88.4 (81.5-95.7)	102.9 (22.5)	99.6 (86.2-118)
[205,2272]	3300 (25.0%)	51 (14.2)	50.1 (39.2-62.3)	14.5 (11.2)	12.4 (5.7-18.7)	85.9 (10)	85.5 (79.4-92.5)	104.2 (16.7)	104.6 (91.1-115.8)
**GDP per capita in 2008 (yuan)**									
[6475,13803)	3532 (26.7%)	49 (15.6)	47.5 (36.8-62.3)	11.3 (6.7)	11 (5.1-16.3)	88.3 (11.6)	87.2 (80.3-95.1)	100 (22.2)	96.1 (83.5-116)
[13803,21053)	3072 (23.3%)	55.3 (15.1)	54.9 (43.4-66.6)	14.8 (8.2)	13.4 (9.3-18.8)	87.8 (10.7)	87.2 (80.7-94.4)	105.7 (19)	106.1 (91-118.3)

	Overall (N=13207)	Annual $PM_{2.5}$ (μg/m³)		Annual NO_2 (μg/m³)		Annual O_3 (μg/m³)		Peak season O_3 (μg/m³)	
		Mean (SD)	Median (P25-P75)	Mean (SD)	Median (P25-P75)	Mean (SD)	Median (P25-P75)	Mean (SD)	Median (P25-P75)
[21053,45466)	3330 (25.2%)	55 (17.2)	53.1 (42.1-65.9)	22.7 (12.4)	20.7 (14-28.8)	92.8 (11.9)	92.7 (84.9-100.6)	112.8 (19.6)	112.7 (99.6-125.8)
[45466,80655]	3273 (24.8%)	52.2 (14.2)	49.9 (43.3-57)	27.4 (18.2)	21.3 (16-31.8)	99 (9.6)	100.1 (93.4-105.1)	123.7 (15.3)	123.2 (114.8-132.7)
**Population density in 2008 (per square kilometer)**									
[25.3, 338)	3321 (25.1%)	44.2 (13.2)	43.7 (34.9-51.5)	11.5 (8.6)	9.8 (5.1-15)	85.3 (9.5)	85.2 (79.1-91.6)	93.5 (17.4)	91.4 (82.6-103.9)
[337.6, 532)	3336 (25.3%)	51.2 (13)	50.6 (40.9-61.5)	17.5 (11.2)	14.7 (9.9-21.9)	89 (11.3)	88.5 (81.3-96.9)	107.7 (20.3)	108.6 (92.1-122.2)
[531.7, 764)	3297 (25.0%)	56.1 (16.2)	55.3 (44.3-65.3)	18.4 (9.2)	17.2 (12.8-22.4)	94.4 (10.7)	94.6 (88-101.6)	116.3 (16.1)	116.8 (105.9-126.2)
[764.0,3376]	3253 (24.6%)	59.6 (16.1)	59 (47.2-69.2)	28.7 (18)	22.7 (17.3-33.7)	99.3 (11)	100.2 (92.4-105.9)	124.5 (17.3)	124.1 (114.1-135)
**Season of the last month**									
Spring	2876 (21.8%)	54.9 (15.6)	52.9 (44.4-64.4)	20 (13.6)	17.3 (11.3-24.6)	90.1 (11.8)	90.4 (82-98.7)	107.9 (19.8)	109.4 (92.6-121.9)
Summer	4009 (30.4%)	50.4 (15.4)	48.6 (39-60.9)	18.9 (13.9)	15.8 (10.5-22.6)	93.4 (11.8)	93 (85-101.7)	112.6 (21.4)	114 (97.8-126.2)
Fall	3220 (24.4%)	51.7 (15.8)	49.8 (39.6-62.6)	18.4 (14.2)	15.1 (9.8-22.3)	92.7 (12)	92.3 (84-101)	110.9 (21.9)	112.8 (93.2-126.3)
Winter	3102 (23.5%)	54.9 (15.8)	53.3 (43.9-64.9)	18.9 (13.1)	16.8 (10.6-23.2)	91.2 (11.6)	91.2 (83.2-99.4)	109.5 (21.3)	111.2 (93.4-124.4)

a. Abbreviations: SD – standard deviation, P25 – percentile 25, P75 – percentile 75, BMI- body mass index, MMSE- Mini-Mental State Examination, ADL - Activities of Daily Living.

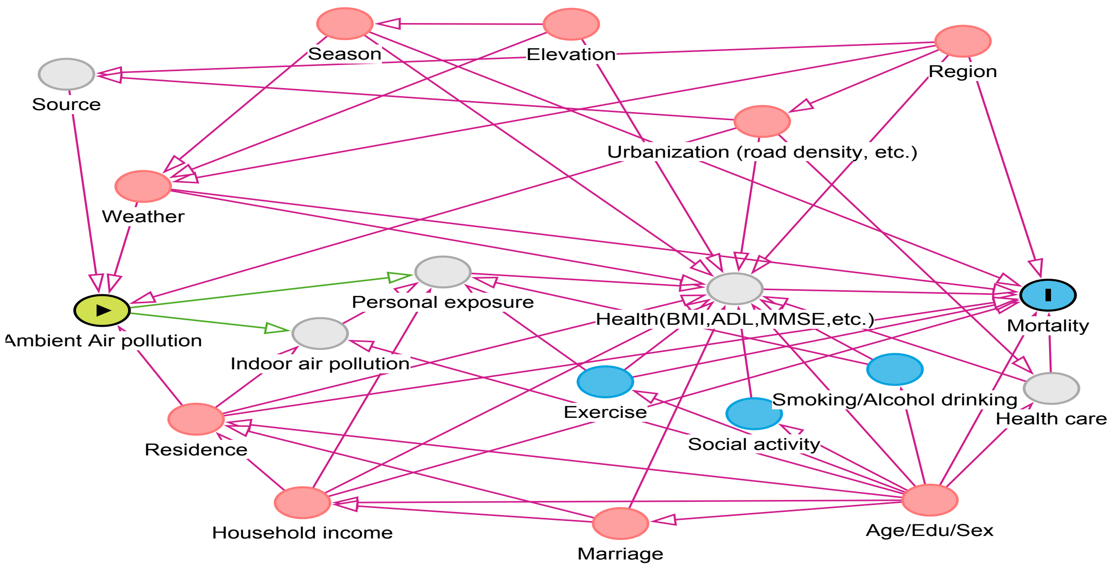

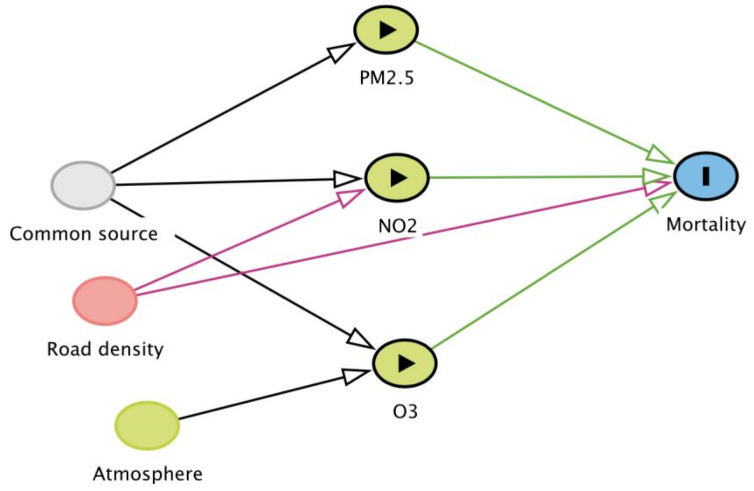

Figure S1. The Directed Acyclic Graph (DAG) of the relationship between ambient air pollution and mortality

Note: We drew the figure using DAGitty v3.1.

Table S3 Association between the two-year and three-year air pollutants' level and mortality risk

Model	Adjustment	Two-year $PM_{2.5}$	Two-year NO_2	Two-year O_3	Three-year $PM_{2.5}$	Three-year NO_2	Three-year O_3
A	**Unadjusted**	1.22 (1.20, 1.23)	1.05 (1.03, 1.06)	0.83 (0.82, 0.85)	1.22 (1.20, 1.23)	1.02 (1.01, 1.04)	0.87 (0.85, 0.89)
B	**Adjusted for age, sex, education, household income, marriage, co-residence, exercise, smoking, alcohol drinking, housework frequency, social activity frequency, residence**	1.13 (1.11, 1.14)	1.03 (1.01, 1.05)	0.90 (0.88, 0.91)	1.13 (1.11, 1.14)	1.01 (0.99, 1.02)	0.93 (0.91, 0.95)
C	**Model B additionally adjusted for region, temperature annual mean, temperature annual SD, elevation, season**	1.21 (1.19, 1.24)	1.03 (1.01, 1.05)	0.87 (0.85, 0.89)	1.21 (1.19, 1.24)	1.00 (0.98, 1.02)	0.91 (0.89, 0.93)
D	**Model C additionally adjusted for road density, city GDP per capita, city population density**	1.22 (1.20, 1.24)	1.13 (1.09, 1.16)	0.86 (0.84, 0.88)	1.22 (1.20, 1.24)	1.06 (1.03, 1.09)	0.90 (0.88, 0.92)
E	**Model D additionally adjusted for BMI, MMSE score, ADL score**	1.22 (1.20, 1.24)	1.12 (1.09, 1.15)	0.86 (0.84, 0.88)	1.22 (1.20, 1.24)	1.05 (1.02, 1.08)	0.90 (0.88, 0.92)

Note. a. Abbreviations: SD – standard deviation, GDP – Gross domestic product, BMI- body mass index, MMSE- Mini-Mental State Examination, ADL - Activities of Daily Living.

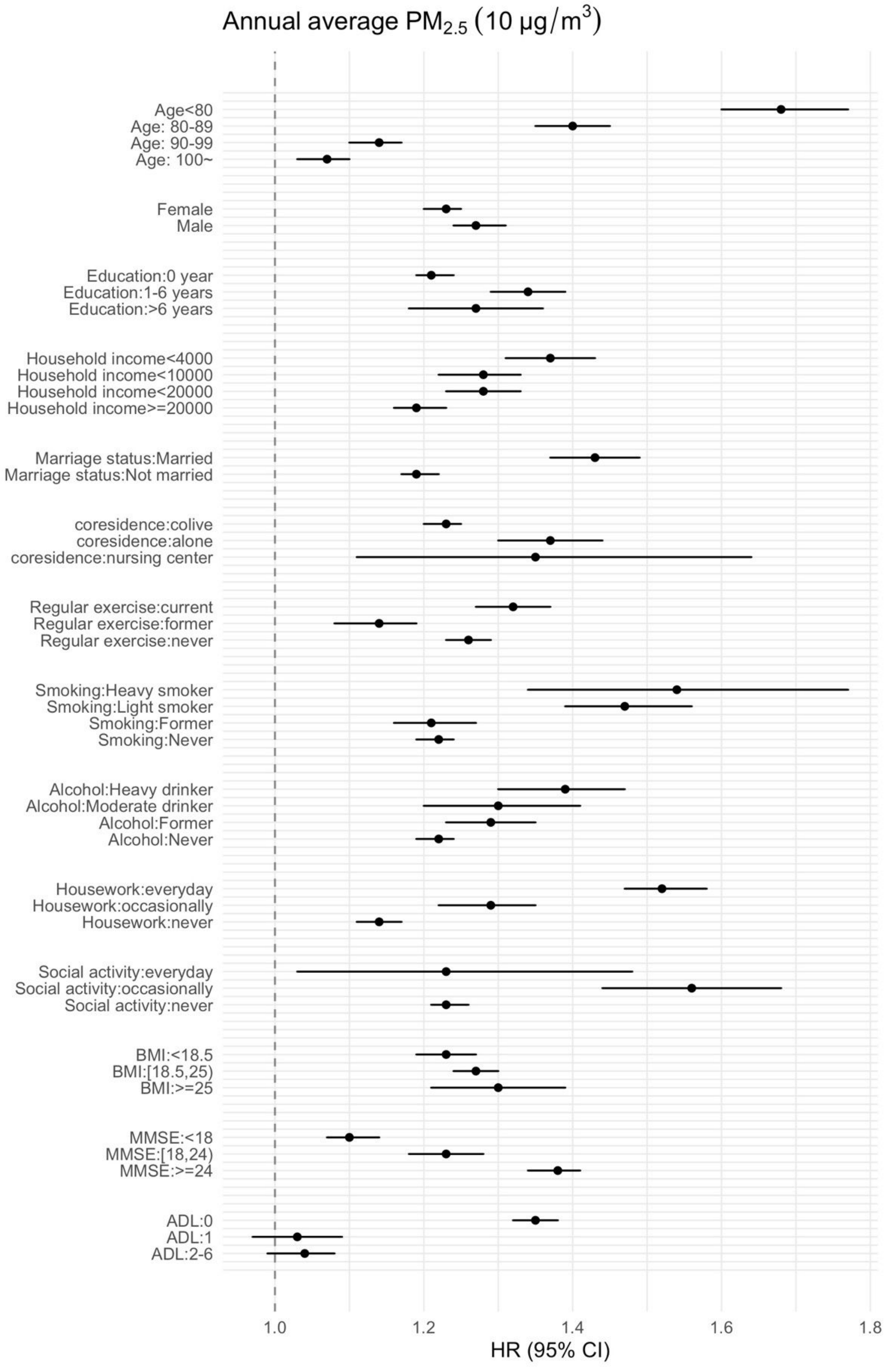
Annual average PM2.5 (10 μg/m3)
Age<80
Age: 80-89
Age: 90-99
Age: 100~
Female
Male
Education:0 year
Education:1-6 years
Education:>6 years
Household income<4000
Household income<10000
Household income<20000
Household income>=20000
Marriage status:Married
Marriage status:Not married
coresidence:colive
coresidence:alone
coresidence:nursing center
Regular exercise:current
Regular exercise:former
Regular exercise:never
Smoking:Heavy smoker
Smoking:Light smoker
Smoking:Former
Smoking:Never
Alcohol:Heavy drinker
Alcohol:Moderate drinker
Alcohol:Former
Alcohol:Never
Housework:everyday
Housework:occasionally
Housework:never
Social activity:everyday
Social activity:occasionally
Social activity:never
BMI:<18.5
BMI:[18.5,25)
BMI:>=25
MMSE:<18
MMSE:[18,24)
MMSE:>=24
ADL:0
ADL:1
ADL:2-6
1.0
1.2
1.4
1.6
1.8
HR (95% CI)

(a)

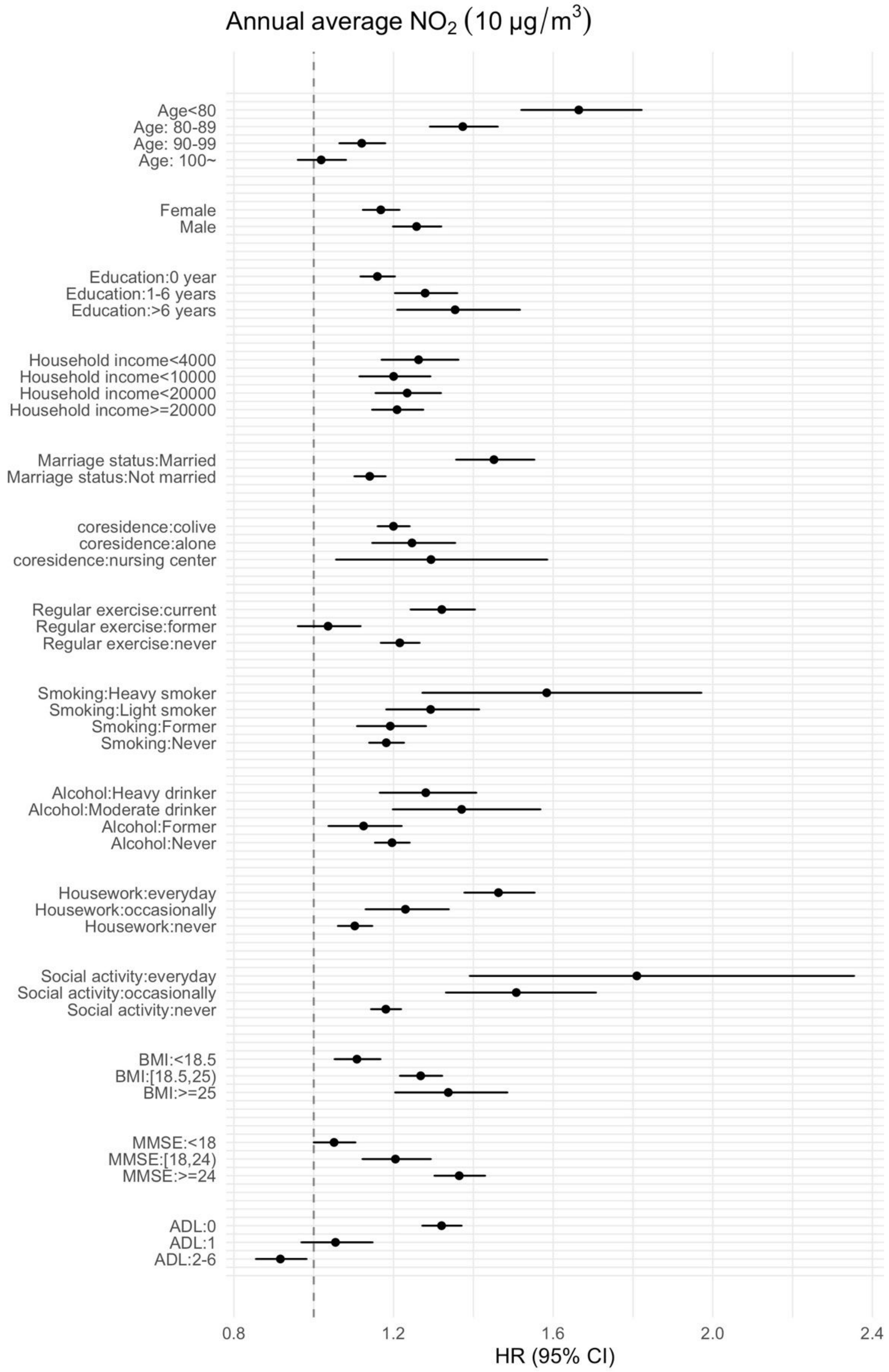
Annual average NO_2 (10 μg/m^3)
Age<80
Age: 80-89
Age: 90-99
Age: 100~
Female
Male
Education:0 year
Education:1-6 years
Education:>6 years
Household income<4000
Household income<10000
Household income<20000
Household income>=20000
Marriage status:Married
Marriage status:Not married
coresidence:colive
coresidence:alone
coresidence:nursing center
Regular exercise:current
Regular exercise:former
Regular exercise:never
Smoking:Heavy smoker
Smoking:Light smoker
Smoking:Former
Smoking:Never
Alcohol:Heavy drinker
Alcohol:Moderate drinker
Alcohol:Former
Alcohol:Never
Housework:everyday
Housework:occasionally
Housework:never
Social activity:everyday
Social activity:occasionally
Social activity:never
BMI:<18.5
BMI:[18.5,25)
BMI:>=25
MMSE:<18
MMSE:[18,24)
MMSE:>=24
ADL:0
ADL:1
ADL:2-6
0.8
1.2
1.6
2.0
2.4
HR (95% CI)

(b)

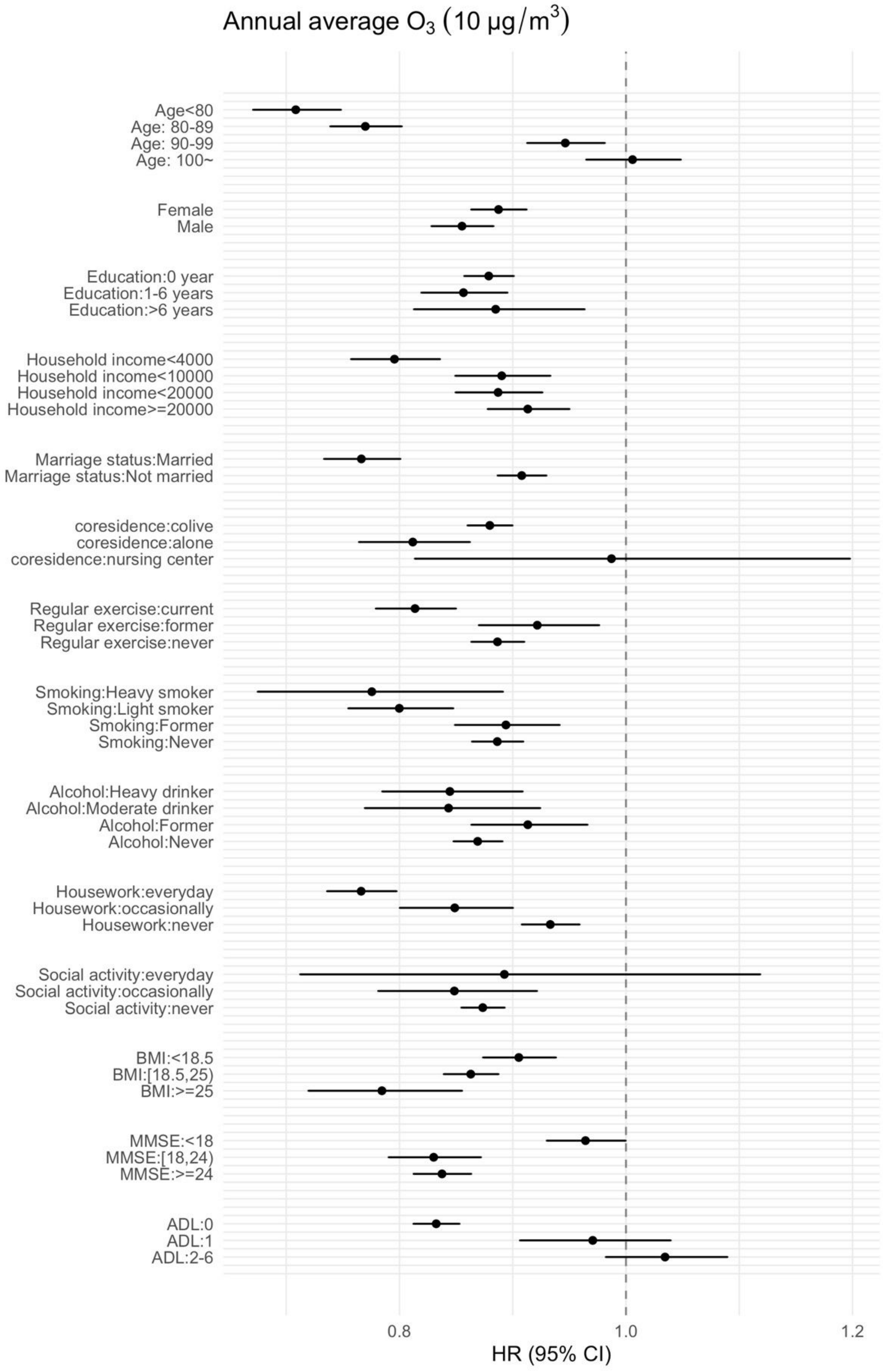

Annual average O_3 ($10\ \mu g/m^3$)
Age<80
Age: 80-89
Age: 90-99
Age: 100~
Female
Male
Education:0 year
Education:1-6 years
Education:>6 years
Household income<4000
Household income<10000
Household income<20000
Household income>=20000
Marriage status:Married
Marriage status:Not married
coresidence:colive
coresidence:alone
coresidence:nursing center
Regular exercise:current
Regular exercise:former
Regular exercise:never
Smoking:Heavy smoker
Smoking:Light smoker
Smoking:Former
Smoking:Never
Alcohol:Heavy drinker
Alcohol:Moderate drinker
Alcohol:Former
Alcohol:Never
Housework:everyday
Housework:occasionally
Housework:never
Social activity:everyday
Social activity:occasionally
Social activity:never
BMI:<18.5
BMI:[18.5,25)
BMI:>=25
MMSE:<18
MMSE:[18,24)
MMSE:>=24
ADL:0
ADL:1
ADL:2-6
0.8
1.0
1.2
HR (95% CI)

(c)

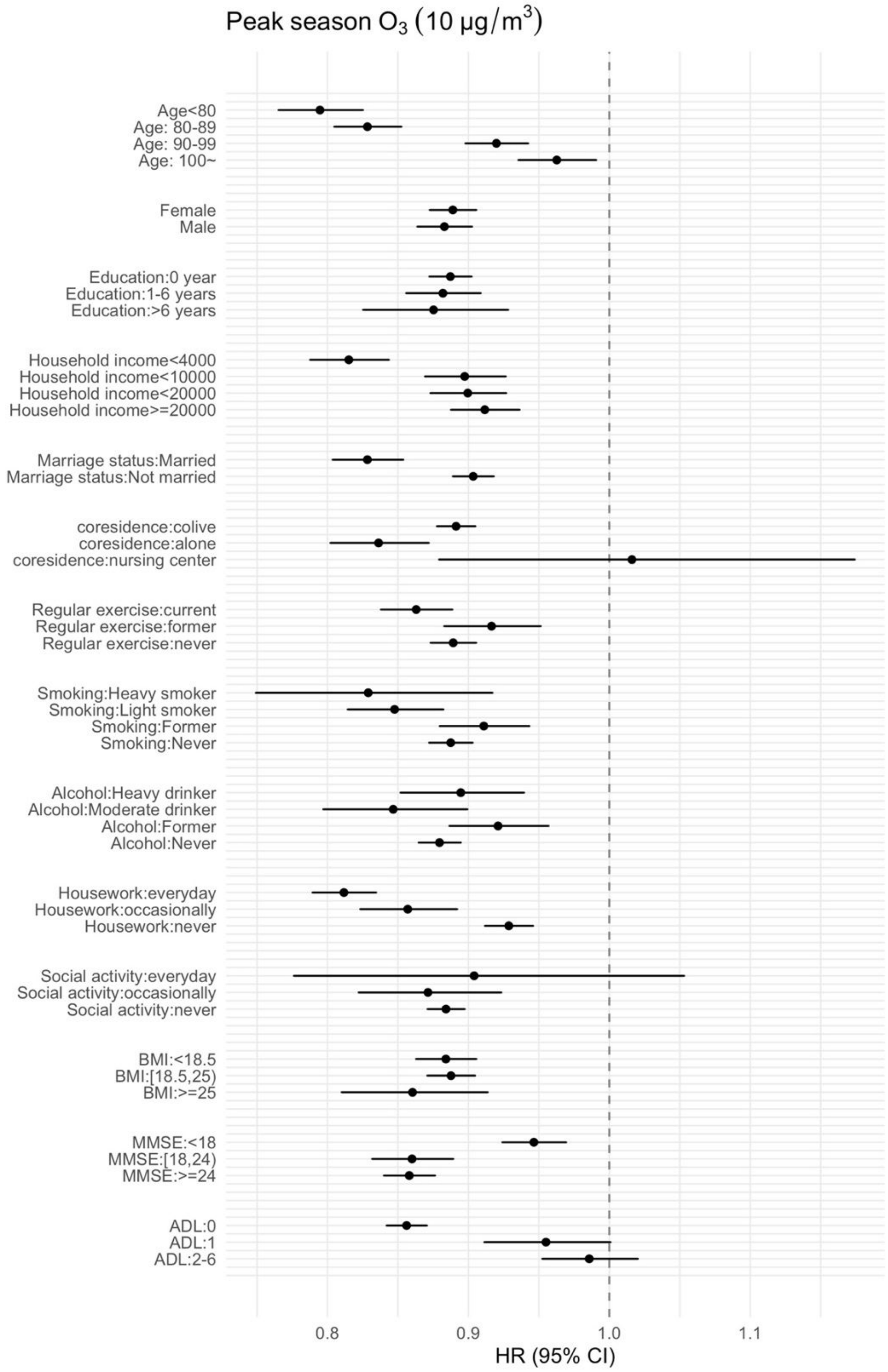

(d)

Figure S2. Association between air pollutants and mortality stratified by individual characteristics

a. Abbreviations: BMI- body mass index, MMSE- Mini-Mental State Examination, ADL - Activities of Daily Living.
b. Unit: Household income – RMB, BMI – kg/m^2

Extended Discussion

Separate association between air pollutants and mortality

The latest review on $PM_{2.5}$ and mortality found the combined effect estimate across 25 studies was 1.08 (95% CI:1.06, 1.09) per 10 μg/m^3 increase in $PM_{2.5}$ for natural-cause mortality [2]. It is lower than our estimated HR of $PM_{2.5}$ in all adjusted models. The relatively high $PM_{2.5}$ and older age population may explain this difference. Our result was also higher than the study based on the same cohort [HR 1.08 (95% CI: 1.06,1.09)] [3]. The major difference was that we had a four-year longer follow-up and adjusted for more possible district-level confounders.

There were much fewer long-term NO_2 and O_3 studies on mortality than $PM_{2.5}$. A review in 2020 only included two Chinese studies for NO_2 and no Chinese study for O_3 [4]. It found a 10 μg/m^3 increase in NO_2 was associated with a RR of 1.02 (95% CI: 1.01, 1.04) for mortality from all-causes [4]. The certainty of the evidence was moderate for NO_2 and all-cause mortality. Two previous studies in China did not find a significant association or an HR of approximately 1.0 for NO_2 and all-cause mortality [5,6]. The HR (95% CI) per 10 μg/m^3 increase of NO_2 in the present study was higher than this. It was 1.06 (1.04, 1.08), and became much stronger after adjusting for road density, GDP per capita, and population density [1.21 (1.17, 1.25)]. A recent long-term NO_2 national study in China reported a similar HR of 1.22 (95% CI: 1.10, 1.35) per 10 μg/m^3 increase in NO_2 and mortality adjusted for annual temperature mean [7].

Pooled results based on nine studies showed no significant association between increased all-year O_3 exposure and all-cause mortality as HR being 0.97 (95% CI: 0.93, 1.02) per 10 μg/m^3 with a large heterogeneity [4]. We found that higher O_3 was associated with lower mortality in the total population.

References

(1) Millwood, I. Y.; Li, L.; Smith, M.; Guo, Y.; Yang, L.; Bian, Z.; Lewington, S.; Whitlock, G.; Sherliker, P.; Collins, R.; Chen, J.; Peto, R.; Wang, H.; Xu, J.; He, J.; Yu, M.; Liu, H.; Chen, Z. Alcohol Consumption in 0.5 Million People from 10 Diverse Regions of China: Prevalence, Patterns and Socio-Demographic and Health-Related Correlates. *Int. J. Epidemiol.* **2013**, *42* (3), 816–827. https://doi.org/10.1093/ije/dyt078.

(2) Chen, J.; Hoek, G. Long-Term Exposure to PM and All-Cause and Cause-Specific Mortality: A Systematic Review and Meta-Analysis. *Environ. Int.* **2020**, *143*, 105974. https://doi.org/10.1016/j.envint.2020.105974.

(3) Li, T.; Zhang, Y.; Wang, J.; Xu, D.; Yin, Z.; Chen, H.; Lv, Y.; Luo, J.; Zeng, Y.; Liu, Y.; Kinney, P. L.; Shi, X. All-Cause Mortality Risk Associated with Long-Term Exposure to Ambient PM2·5 in China: A Cohort Study. *Lancet Public Heal.* **2018**, *3* (10), e470–e477. https://doi.org/10.1016/S2468-2667(18)30144-0.

(4) Huangfu, P.; Atkinson, R. Long-Term Exposure to NO2 and O3 and All-Cause and Respiratory Mortality: A Systematic Review and Meta-Analysis. *Environ. Int.* **2020**, *144*, 105998. https://doi.org/10.1016/j.envint.2020.105998.

(5) Chen, X.; Zhang, L. wen; Huang, J. ju; Song, F. ju; Zhang, L. ping; Qian, Z. min; Trevathan, E.; Mao, H. jun; Han, B.; Vaughn, M.; Chen, K. xin; Liu, Y. min; Chen, J.; Zhao, B. xin; Jiang, G. hong; Gu, Q.; Bai, Z. peng; Dong, G. hui; Tang, N. jun. Long-Term Exposure to Urban Air Pollution and Lung

Cancer Mortality: A 12-Year Cohort Study in Northern China. *Sci. Total Environ.* **2016**, *571* (22), 855–861. https://doi.org/10.1016/j.scitotenv.2016.07.064.

(6) Yang, Y.; Tang, R.; Qiu, H.; Lai, P. C.; Wong, P.; Thach, T. Q.; Allen, R.; Brauer, M.; Tian, L.; Barratt, B. Long Term Exposure to Air Pollution and Mortality in an Elderly Cohort in Hong Kong. *Environ. Int.* **2018**, *117*, 99–106. https://doi.org/10.1016/j.envint.2018.04.034.

(7) Wang, Y.; Luo, S.; Wei, J.; Yang, Z.; Hu, K.; Yao, Y.; Zhang, Y. Ambient NO2 Exposure Hinders Long-Term Survival of Chinese Middle-Aged and Older Adults. *Sci. Total Environ.* **2023**, *855* (2), 158784. https://doi.org/10.1016/j.scitotenv.2022.158784.
